# Chromosomal-level reference genome assembly of the North American wolverine (*Gulo gulo luscus*): a resource for conservation genomics

**DOI:** 10.1093/g3journal/jkac138

**Published:** 2022-06-08

**Authors:** Si Lok, Timothy N H Lau, Brett Trost, Amy H Y Tong, Richard F Wintle, Mark D Engstrom, Elise Stacy, Lisette P Waits, Matthew Scrafford, Stephen W Scherer

**Affiliations:** The Centre for Applied Genomics, Peter Gilgan Centre for Research and Learning, The Hospital for Sick Children, Toronto, ON M5G 0A4, Canada; Program in Genetics and Genome Biology, The Hospital for Sick Children, Toronto, ON M5G 0A4, Canada; The Centre for Applied Genomics, Peter Gilgan Centre for Research and Learning, The Hospital for Sick Children, Toronto, ON M5G 0A4, Canada; Program in Genetics and Genome Biology, The Hospital for Sick Children, Toronto, ON M5G 0A4, Canada; The Centre for Applied Genomics, Peter Gilgan Centre for Research and Learning, The Hospital for Sick Children, Toronto, ON M5G 0A4, Canada; Program in Genetics and Genome Biology, The Hospital for Sick Children, Toronto, ON M5G 0A4, Canada; Donnelly Centre for Cellular and Biomolecular Research, University of Toronto, ON M5S 3E1, Canada; The Centre for Applied Genomics, Peter Gilgan Centre for Research and Learning, The Hospital for Sick Children, Toronto, ON M5G 0A4, Canada; Program in Genetics and Genome Biology, The Hospital for Sick Children, Toronto, ON M5G 0A4, Canada; Department of Natural History, Royal Ontario Museum, Toronto, ON M5S 2C6, Canada; Environmental Science Program, University of Idaho, Moscow, ID 83844, USA; Wildlife Conservation Society, Arctic Beringia, Fairbanks, AK 99709, USA; Department of Fish and Wildlife, University of Idaho, Moscow, ID 83844, USA; Wildlife Conservation Society Canada, Thunder Bay, ON P7A 4K9, Canada; The Centre for Applied Genomics, Peter Gilgan Centre for Research and Learning, The Hospital for Sick Children, Toronto, ON M5G 0A4, Canada; Program in Genetics and Genome Biology, The Hospital for Sick Children, Toronto, ON M5G 0A4, Canada; McLaughlin Centre, University of Toronto, Toronto, ON M5G 0A4, Canada; Department of Molecular Genetics, Faculty of Medicine, University of Toronto, ON M5S 1A8, Canada

**Keywords:** wolverine, *Gulo gulo*, *Gulo gulo luscus*, conservation genomics, aggression genes, innate immunity, coronavirus, SARS-CoV-2, long-reads, chromosomal-level assembly

## Abstract

We report a chromosomal-level genome assembly of a male North American wolverine (*Gulo gulo luscus*) from the Kugluktuk region of Nunavut, Canada. The genome was assembled directly from long-reads, comprising: 758 contigs with a contig N50 of 36.6 Mb; contig L50 of 20; base count of 2.39 Gb; and a near complete representation (99.98%) of the BUSCO 5.2.2 set of 9,226 genes. A presumptive chromosomal-level assembly was generated by scaffolding against two chromosomal-level Mustelidae reference genomes, the ermine and the Eurasian river otter, to derive a final scaffold N50 of 144.0 Mb and a scaffold L50 of 7. We annotated a comprehensive set of genes that have been associated with models of aggressive behavior, a trait which the wolverine is purported to have in the popular literature. To support an integrated, genomics-based wildlife management strategy at a time of environmental disruption from climate change, we annotated the principal genes of the innate immune system to provide a resource to study the wolverine’s susceptibility to new infectious and parasitic diseases. As a resource, we annotated genes involved in the modality of infection by the coronaviruses, an important class of viral pathogens of growing concern as shown by the recent spillover infections by severe acute respiratory syndrome coronavirus-2 to naïve wildlife. Tabulation of heterozygous single nucleotide variants in our specimen revealed a heterozygosity level of 0.065%, indicating a relatively diverse genetic pool that would serve as a baseline for the genomics-based conservation of the wolverine, a rare cold-adapted carnivore now under threat.

## Teaser

The present work is the first long-read and chromosomal-level assembly of the wolverine, a rare cold adapted carnivore that has become an iconic symbol of the North American wilderness, now under threat. To support a future integrated, genomics-based wildlife management strategy at a time of environmental disruption from climate change, we annotated the principal genes of the innate immune system to provide a resource to study the wolverine’s susceptibility to new infectious and parasitic diseases.

## Introduction

**Fig. 1.**
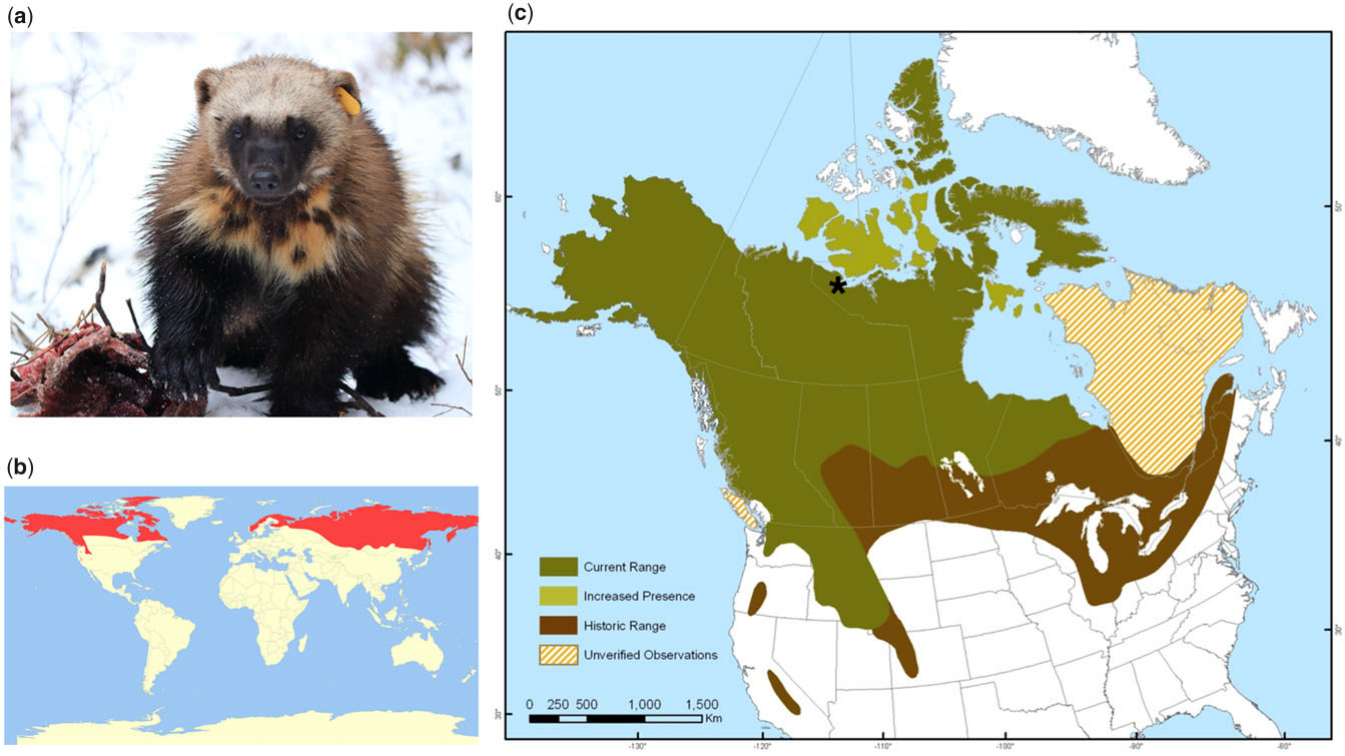
Wolverine distribution. a) A female wolverine monitored in Red Lake, Ontario, as a part of a research study led by Wildlife Conservation Society Canada. The wolverine has an ear tag to allow researchers to identify the individual when caught on camera or in live traps. The researchers have also attached a Global Positioning System (GPS) collar to track the wolverine’s movements relative to extensive commercial mining and forestry in the area. Wolverines were baited to live traps and camera stations with beaver carcasses donated by local trappers. (Photo courtesy of Liam Cowan). b) Pan-arctic distribution of wolverine. World-wide range of the wolverine is indicated in red (Map courtesy of Oona Räisänen, International Union for Conservation of Nature, https://commons.wikimedia.org/wiki/File:Gulo_gulo_distribution.svg). c) Wolverine population in North America (Map courtesy of COSEWIC 2014, ©Her Majesty the Queen in Right of Canada, 2014, Catalog No. CW69-14/329-2014E-PDF, ISBN 978-1-100-23964-4). The Kugluktuk (Coppermine) region of Nunavut (67°48′43″N 115°05′05″W) where our specimen (Royal Ontario Museum tissue archive no FN33715-3; NCBI BioSample SAMN16725402) originated is denoted by an asterisk (*).

The wolverine (*Gulo gulo* spp.) is a generalist carnivore and the largest terrestrial member of the weasel family (Mustelidae), inhabiting circumpolar mountains, tundra, and the lowland boreal forests (Copeland and Whitman 2003) (Fig. 1, a and b). The wolverine’s utilization of deep and long-lived snowpack for denning structures (Copeland *et al.* 2010) and food-caching locations (van der Veen *et al.* 2020) are behavioral adaptations to this harsh climatic niche. The wolverine is also physiologically adapted to cold and snowy environments with stocky build and thick fur for heat retention, wide paws and plantigrade stride to walk on top of snow, and skull characteristics, including an enlarged sagittal crest and zygomatic arch, muscular head, and strong dentition to enable them to crush through frozen bone and meat (Copeland and Whitman 2003). The latter ability is reflective of its scientific species name derived from the Latin “gula” and “gulosus,” which translate to “throat” and “gluttonous,” respectively. One food source is carrion, where the wolverine stands out as dominant among the mesocarnivores, as exemplified by their low vigilance at carcass sites, indicating that they do not consider wolves or other carnivore species as serious threats (Klauder *et al.* 2021). Due to their reclusive nature and large home range (often more than 1,000 km^2^ per adult male), wolverines are seldom seen and have become an iconic symbol and elusive spirit of the North American wilderness (Bonamy *et al.* 2020).

Wolverines are taxonomically divided into the North American (new world) (*G.* *gulo luscus*) and the Eurasian (old world) (*G.* *gulo gulo*) subspecies (Kurten and Rausch 1959; Copeland and Whitman 2003). Wolverines are considered a species of Least Concern globally because of large populations throughout northern North America and Asia (Abramov 2016). However, populations are declining in many regions because of habitat loss and increased mortality associated with poaching, trapping, and hunting, albeit at a relatively slower rate than other sensitive species (Laliberte and Ripple 2004; Abramov 2016). Anthropogenic mortality remains a major conservation concern for wolverine populations worldwide (Krebs *et al.* 2004; Persson *et al.* 2009). In North America, wolverine distribution has declined by nearly 40% since European settlement (Laliberte and Ripple 2004). Climate projections and distribution modeling show that dispersal corridors and suitable habitat at the southern extent of their range could be lost, leading to further population isolation (McKelvey *et al.* 2011; Barsugli *et al.* 2020). However, wolverine populations in western and northern Canada are relatively stable, with increased sightings observed in Banks, Victoria, and other islands of the Canadian Arctic Archipelago (Species at Risk Committee, 2014). In contrast, populations in eastern Canadian provinces are extirpated or endangered (COSEWIC 2014) (Fig. 1c). Wolverines in the contiguous United States are of conservation concern and were considered by the U.S. Fish and Wildlife Services in 2013 for threatened species status under the Endangered Species Act. There, wolverines reside in isolated mountain ranges (Lukacs *et al.* 2020) dependent on gene flow from southwest Canada for population persistence (Cegelski *et al.* 2006; McKelvey *et al.* 2014).

Conservation and management plans have been produced by environmental groups and the Canadian and American governments to monitor and protect the wolverine (COSEWIC 2014; Idaho Department of Fish and Game 2014; Environment Canada 2016). An important component of these plans is the availability of mitochondrial DNA (Wilson *et al.* 2000; Tomasik and Cook 2005) and microsatellite markers (Supplementary Table 1 and references therein). These markers have been used for monitoring wolverine gene flow (Schwartz *et al.* 2007; Gervasi *et al.* 2015), habitat connectivity (Schwartz *et al.* 2009; Sawaya *et al.* 2019; Balkenhol *et al.* 2020), reproductive success (Hedmark *et al.* 2007), range contraction and expansion (McKelvey *et al.* 2014; Krejsa et al. 2021), population size and distribution (Kortello *et al.* 2019; Mowat *et al.* 2020), effective population size (Schwartz *et al.* 2009; Lansink *et al.* 2020), and population structure and diversity (Kyle and Strobeck 2002; Cegelski *et al.* 2006). However, the majority of the microsatellite markers were originally developed for other members of Mustelidae, and only a small subset of them have been tested and shown to be informative in wolverine. Moreover, it was found that the low levels of diversity in the existing sets of microsatellite and mitochondrial loci limited their usefulness (Walker *et al.* 2001; Väli *et al.* 2008). For improved resolution of genetic variations and the ability to investigate adaptive potential, a large set of neutral and adaptive markers is needed. Hence, there is an unmet need for additional genomic markers in wolverine conservation and management.

A significant advance was the recent generation of a draft genome assembly for the Eurasian wolverine (Ekblom *et al.* 2018). While this assembly was relatively fragmented, typical of those generated from short-reads, it provided an important first glimpse of the Eurasian wolverine genome and enabled “reference-assisted whole genome resequencing” (referred to as “resequencing”), leading to the development of genome-wide informative genotyping arrays for use in low-cost population surveys (Ekblom *et al.* 2021). Here, we report a chromosomal-level assembly of the North American wolverine. In addition to provide a more tailored reference genome for use in resequencing of North American wolverines, the contiguity offered by this assembly provides insight into the genomic architecture of the wolverine and the genetic discrimination between old and new world subspecies.

## Materials and methods

### Sample verification and DNA extraction

**Fig. 2.**
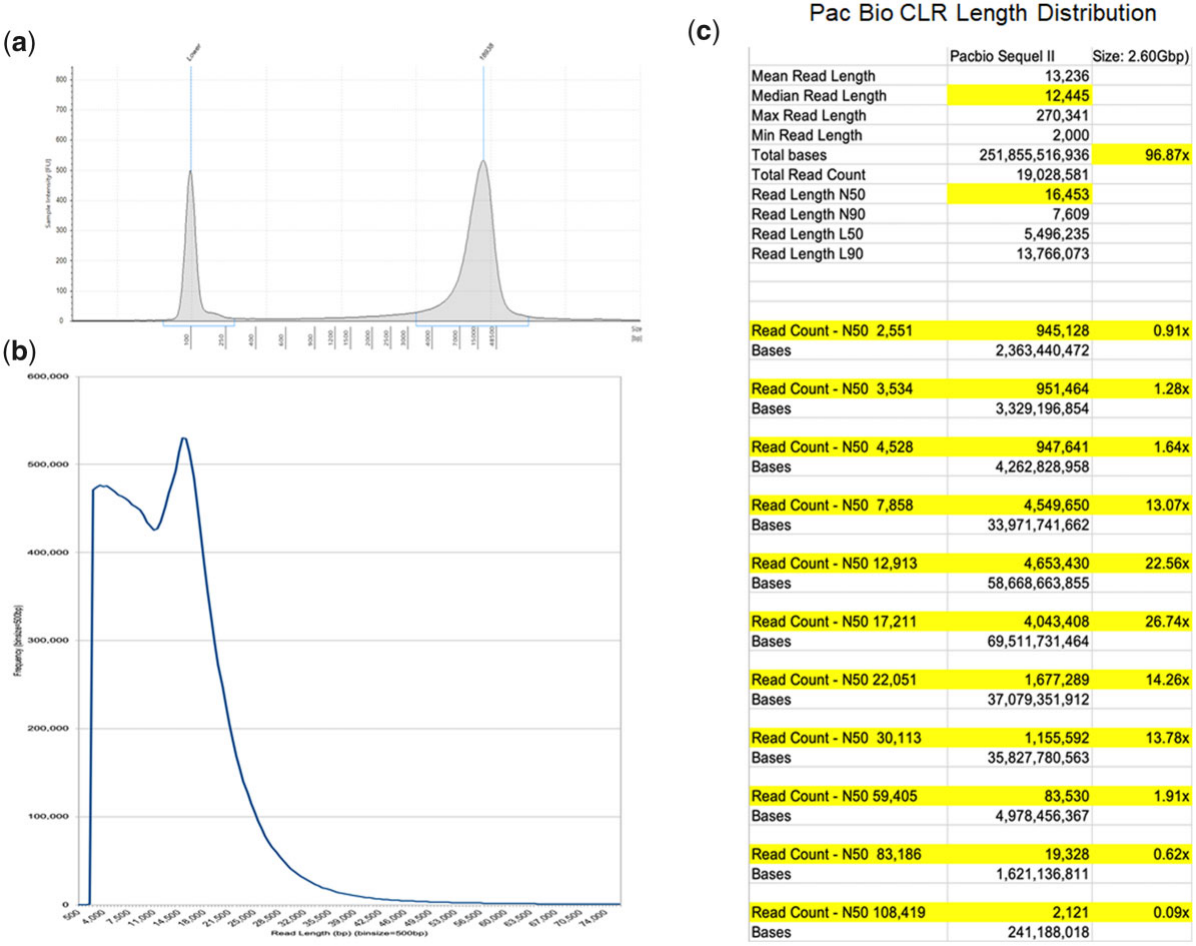
Wolverine genomic DNA profile and length distribution of sequence reads (NCBI BioSample SAMN16725402). a) Length distribution profile of wolverine genomic DNA on the Agilent TapeStation (Agilent). Based on the internal 100 bp calibration marker, genomic DNA used in the study has a peak length of 18.9 kb. b) Length profile of 19,028,581 CLRs generated on the Sequel II sequencer (Pacific Biosciences). For genome assembly, reads <2 kb in length were discarded (see *Materials and methods*). The remaining reads have a read length N50 of 16,453 bp, comprising 251.8 Gb of sequences, which represented an estimated 96.9x coverage of the wolverine genome. c) The side panel shows the estimated genomic coverage of the wolverine genome by reads of different lengths. The read-length N50 value for each length category is as indicated.

Genomic DNA was extracted from frozen kidney tissue of an archival male North American wolverine specimen (Royal Ontario Museum tissue archive no FN33715-3; NCBI BioSample SAMN16725402), using the MagAttract HMW DNA kit (Qiagen, Hilden, Germany). The resulting DNA has a peak length of 18.9 kb on the Agilent TapeStation (Agilent, Santa Clara, CA), and 260 nm/280 nm and 260 nm/230 nm absorbance ratios of 1.95 and 2.45, respectively (Fig. 2a). DNA was quantified for library construction by fluorometry using the Qubit DNA HS Assay (ThermoFisher, Waltham, MA). Species identity of the specimen was verified by matching a segment of the cytochrome oxidase 1 (*COX1*) gene from the specimen’s assembled mitochondrion against the Barcode of Life Database (http://www.boldsystems.org). The top seven hits in the database were from wolverines collected in North America at 100% match. The next six hits were from Eurasian wolverines at 99.8% match. Using human *SRY* as a query, the sex of our wolverine specimen was confirmed as a male by the annotation of the Y chromosome-specific gene, *SRY* (Sex-determining Region Y), in the assembled genome. As a negative control for BLAST search, we did not find *SRY* in the female Eurasian wolverine assembly (Ekblom *et al.* 2018; Gulo_2.2_annotated) using the same search threshold.

### Genome sequencing and preassembly filtering

Long-reads for assembling the wolverine genome were produced from a library prepared from 5 µg of unsheared genomic DNA using the Pacific Biosciences’ (PacBio) Express template prep kit version 2.0 (Pacific Biosciences, Menlo Park, CA) followed by a > 14 kb post-library sizing step on the BluePippin system (Sage Science, Beverly, MA). The resulting size-selected library was sequenced on three flow cells on a Sequel II sequencer (Pacific Biosciences) in the continuous long read (CLR) mode with a 15-h movie acquisition time. Raw reads were processed with PacBio’s P filter to remove low-quality reads and adaptor sequences. The longest sub-read was selected from each productive zero-mode waveguide of the Sequel II flow cell. Only sub-reads are used in the present study, and are therein referred to as “reads” in this manuscript. Reads of <2 kb were discarded. After processing, 19,028,581 reads remained with a read length N50 of 16,453 bp, comprising 251.8 Gb of sequences. Figure 2c shows the genomic coverage at different read lengths, as indicated by the N50 values for each read length category.

Short-reads for polishing the assembled genome were produced from 700 ng of genomic DNA that was randomly fragmented to a size of 600-700 bp using a Covaris LE220 Focused Ultrasonicator (Covaris, Woburn, MA). Library construction was carried out in accordance with the Illumina TruSeq DNA PCR-Free protocol (Version 1000000039279v00) (Illumina, San Diego, CA). Sequencing was performed on two lanes of the HiSeq-X sequencer (Illumina), producing 2 × 150 bp paired-end reads. Reads were trimmed to retain only the most accurate portion. Only Illumina sequences with quality scores better than Q35 were used. To achieve this level of accuracy, typically the first 10 bases were discarded in read 1 and read 2; the last 20 bases were discarded in read 1; and the last 30 bases were discarded in read 2. Adaptor sequences, if any, were removed. After trimming, 783 million paired-end reads remained, comprising 180 Gb of sequences. Using a size for the wolverine genome of 2.6 Gb, the present study generated 96.9x coverage in processed PacBio CLRs and 69.3x coverage in trimmed Illumina paired-end reads.

### Size estimation of the wolverine genome

The size of the wolverine genome was estimated by tabulating k-mer frequencies of 783 million Illumina reads using preQC (Simpson 2014) and Jellyfish (https://github.com/gmarcais/Jellyfish), providing a genome size of 2.33 and 2.48 Gb, respectively. For the calculation of genome coverage and related assembly metrics, we used a genome size of 2.6 Gb, which is the mean value from k-mer tabulation and the estimated genome size of 2.7–2.8 Gb recently reported for the Eurasian wolverine (Ekblom *et al.* 2018).

### *De novo* genome assembly and reference-assisted chromosomal scaffolding

**Fig. 3.**
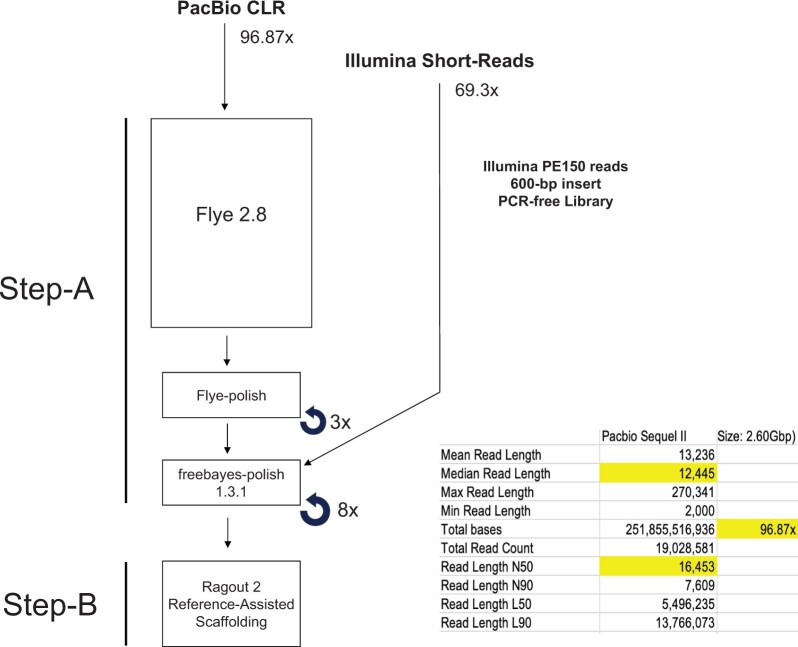
Long-read assembly workflow. Input DNA is described in the left panel. Step-A: CLRs (Pacific Biosciences) were assembled using Flye 2.8 (Kolmogorov *et al.* 2019). To resolve potential misassemblies and other errors, the primary assembly was polished three times with Flye-polish (Kolmogorov *et al.* 2019) using the same CLRs used to construct the primary assembly. To correct residual sequencing errors after the Flye-polishing step, the assembly was subjected to eight rounds of polishing with high-quality trimmed Illumina short-reads (69.3x genome coverage) using freebayes (https://github.com/freebayes/freebayes). The final assembly after polishing with freebayes 1.3.1 was designated Gulo_gulo_luscus_F-V1.0 (WGS Accession: JAJAGD000000000). Step-B: Ragout 2 (Kolmogorov *et al.* 2018) was used to scaffold the wolverine assembly from Step-A against the two available chromosomal-level assemblies in the Mustelidae [*Mustela erminea* (mMusErm1.Pri) and *Lutra lutra* (mLutLut1.2)], and the dingo assembly (*Canis lupus* *dingo*) (UNSW_AlineDingo_1.0). The final assembly after scaffolding was designated Gulo_gulo_luscus_R-V1.0 (WGS Accession: JAJHUB000000000).

The two-step assembly workflow for the wolverine genome is depicted in Fig. 3. In Step-A, genome assembly was carried out by a simple and direct strategy from uncorrected PacBio reads (96.9x genome coverage) using Flye 2.8, which was designed to assemble error-prone reads (Kolmogorov *et al.* 2019). To resolve potential misassemblies and other errors, the primary assembly was polished three times with Flye-polish (Kolmogorov *et al.* 2019) using the same PacBio CLRs used to construct the primary assembly. To correct remaining PacBio sequencing errors after the Flye-polishing step, the resulting assembly was subjected to eight rounds of additional polishing with high-quality trimmed Illumina short-reads (69.3x genome coverage) using freebayes 1.3.1 (https://github.com/freebayes/freebayes). The polished assembly was inspected by the alignment of PacBio reads (96.9x genome coverage), and a short-read assembly, designated Gulo_gulo_luscus_A-V1.0, assembled from 69.3x coverage of Illumina short-reads using ABySS 2.1.5 (parameters k = 79, kc = 3) (Jackman *et al.* 2017). Regions in the polished assembly that were supported by fewer than five PacBio CLRs (17 such regions in the assembly) were manually inspected and curated. The final assembly after this inspection step was designated Gulo_gulo_luscus_F-V1.0 (WGS Accession: JAJAGD000000000).

In Step-B, Ragout 2 (Kolmogorov *et al.* 2018) was used to scaffold the wolverine assembly from Step-A against the two available chromosomal-level assemblies in the Mustelidae [ermine (*Mustela erminea*) (mMusErm1.Pri) and Eurasian river otter (*Lutra lutra*) (mLutLut1.2)], and the dingo (*Canis lupus* *dingo*) (UNSW_AlineDingo_1.0). Reference-assisted scaffolding was carried out conservatively in which we disabled Ragout 2’s ability to break contigs in its effort to fit the reference genomes during the scaffolding process. In situations where a wolverine contig disagreed with one or more reference genomes, the contig was left intact. The final assembly after scaffolding was designated Gulo_gulo_luscus_R-V1.0 (WGS Accession: JAJHUB000000000).

### Sequence contamination in assembly

To assess potential microbial and other environmental DNA contaminants in our assembly, the Gulo_gulo_luscus_F-V1.0 assembly was divided into 478,025 nonoverlapping 5 kb windows. Each window was searched using BLAST (Altschul *et al.* 1990) against the nonredundant NCBI Nucleotide (nt) Database (Version: 2020 November 19) (https://ftp.ncbi.nlm.nih.gov/blast/db/) (see Supplementary Fig. 1). The nucleotide collection comprises GenBank, EMBL, DDBJ, PDB, RefSeq sequences, but excludes EST, STS, GSS, WGS, TSA, patent sequences, as well as phase 0, 1, and 2 HTGS sequences and sequences longer than 100 Mb. The resulting BLAST results were tabulated using a series of five sequential filters of decreasing phylogenetic distance from the wolverine, comprising: (1) the genome assemblies of the ermine (*M.* *erminea*; GCF_009829155.1) and Eurasian river otter (*L.* *lutra*; GCA_902655055.2); (2) other members of Mustelidae; (3) non-Mustelidae carnivores; (4) organisms outside of carnivora; and (5) a collection of lineages, (a) through (l), each defined by a (group of) Taxonomy Name(s) in NCBI's Taxonomy Database. The lineages in the fifth filter comprised: (5a) Bacteria = Bacteria (NCBI: txid2); (5b) Protist = Eukaryota (NCBI: txid759) minus [Viridiplantae (NCBI: txid33090), Rhodophyta (NCBI: txid2763), Metazoa (NCBI: txid33208), and Fungi (NCBI: txid4751)]; (5c) Plants = Viridiplantae (NCBI: txid33090); (5d) Fungi = Fungi (NCBI: txid751); (5e) Invertebrates = Metazoa (NCBI: txid33208) minus Vertebrata (NCBI: txid7742); (5f) Fishes = Actinopterygii (NCBI: txid7898); (5g) Amphibians = Amphibia (NCBI: txid8292); (5h) Mammals = Mammalia (NCBI: txid40674); (5i) Reptiles = Sauropsida (NCBI: txid8457) minus Aves (NCBI: txid8782); (5j) Birds = Aves (NCBI: txid8782); (5k) Viruses = Viruses (NCBI: txid10239); and (5l) Others = None of the above. The presence of contaminating human DNA in the Gulo_gulo_luscus_F-V1.0 assembly was assessed using a primate specific SINE, *AluY* probe (Longo *et al.* 2011).

### Mitochondrial genome assembly

A single contig of 16,556 bp in the Gulo_gulo_luscus_F-V1.0 assembly was recognized as a presumptive full-length circular mitochondrial genome using human mitochondrion sequence NC_012920.1 as a BLAST query. A mitochondrial genome was also independently assembled from 7.8 million trimmed Illumina paired-end reads with ABySS 2.1.5 (Jackman *et al.* 2017) (parameters k = 97, kc = 4) to yield a single prominent contig of 16,570 bp. Alignment of this contig with the 16,556 bp full-length mitochondrial genome assembled from long-reads revealed that the two contigs were identical except for the presence of a 14 bp duplicated sequence at the termini of the contig assembled from Illumina short-reads. This duplicated sequence is a part of the repetitive region of the mitochondrial D-loop. It is likely this duplication was artifactually generated in the short-read assembly due to the inability of short-reads to resolve this region. Removal of one copy of the 14-base duplicated sequence and circularization of the contig yielded a full-length mitochondrion genome of 16,556 bp identical to that assembled independently from long-reads.

### BUSCO analysis and exon-level gene annotation

The completeness of our Gulo_gulo_luscus_F-V1.0 assembly was qualitatively assessed using BUSCO 5.2.2 (Benchmarking Universal Single Copy Orthologs; mammalia_odb10) (Simão *et al.* 2015), which provided the status of the 9,225 genes in the current BUSCO gene set. Each BUSCO gene was designated by the program as complete, fragmented, or missing, in the wolverine assembly. Exon-level annotation of selected wolverine genes was accomplished using orthologous human and mouse exon probes (NCBI RefSeq). Starting from a seed exon identified on the assembly, typically having the highest BLAST score amongst the exon queries for that gene, we scanned up-stream and down-stream along the contig in incremental 10 kb windows in the search of the adjacent exons. Specific search criteria include the percentage of nucleotide and amino acid sequence identity to the query exon, and the conservation of the reading frame relative to the consensus splice signals. Once an adjacent exon is identified by BLAST, the process is repeated until all the exons for that gene are identified. For a gene to be complete, the full-length polypeptide encoded by the predicted exons must be at least 70% identical to the full-length human or mouse protein reported in RefSeq. Predicted exons in the wolverine are not experimentally verified in the present study. Accessions for wolverine genes annotated in this manner are provided in Supplementary Table 2 and in the annotation accompanying the assemblies.

### Microsatellite markers

PCR primer pairs for the reported microsatellite markers of mustelids (Supplementary Table 1 and references therein) were mapped onto the wolverine Gulo_gulo_luscus_F-V1.0 assembly at four levels of stringency: completely identical, or with one, two, or three single base mismatches. A primer pair was scored as potentially productive for the wolverine, if it passed all of the following sequential filters: (1) primer 1 and primer 2 both mapped to the Gulo_gulo_luscus_F-V1.0 assembly at one of the four aforementioned stringency levels; (2) the members of a mapped primer pair were in an antiparallel configuration; (3) the mapped distance between primers was between 50 and 500 bp (the predicted microsatellite PCR product); and (4) the predicted PCR product had at least 60% sequence identity to the reported amplified product and had other hallmarks consistent with a microsatellite locus, such as the presence of short, tandemly repeated sequences.

### Identification of repetitive DNA in genome

Repetitive elements described in RepBase were identified and tabulated in the wolverine genome using RepeatMasker (Tarailo and Chen 2009).

### Assessment of genetic diversity

Genetic diversity, as exemplified by heterozygous single nucleotide variants (SNVs) and heterozygous small insertions or deletions <50 bp (Indels), was tabulated for the wolverine and compared with the Eurasian river otter and ermine, members of a selected outbred population (human), and two mammalian species reported to have undergone recent genetic bottlenecks, the cheetah (Menott-Raymond and O’Brien 1993) and the Tasmanian devil (Miller *et al.* 2011).

Human SNV and indel calls were made from joint-called VCF files from the expanded 1000 Genomes Project. The files were downloaded from http://ftp.1000genomes.ebi.ac.uk/vol1/ftp/data_collections/1000G_2504_high_coverage/working/20190425_NYGC_GATK/, and the SelectVariants function of GATK 4.1.2.0 was used to extract the variants for a representative member of each major population group: African (AFR) HG01241; Admixed American (AMR) HG01936; East Asian (EAS) HG00675; European (EUR) NA12878; and South Asian (SAS) HG02728. SNV and indel calls from the Eurasian river otter, ermine, cheetah, wolverine, and Tasmanian devil genomes were made according to genome analysis toolkit (GATK) best practices (Van der Auwera *et al.* 2013). Specifically, sequence reads were aligned to the reference genome using BWA 0.7.17-r1198 (Li and Durbin 2009). Duplicate reads were marked using the MarkDuplicates function of GATK 4.1.9.0 (McKenna *et al.* 2010). The average depth of coverage of each sample was calculated using the “depth” function of SAMtools 1.9 (Li and Durbin 2009).

The following source materials were used: Eurasian river otter (GCA_902655055.2, ERR3316171, ERR3316172); ermine (GCA_009829155.1, SRR6963883); Namibian cheetah “Chewbacca” (GCA_001443585.1, SRR2737512, SRR2737513 SRR2737514, SRR2737515, SRR2737516, SRR2737517, SRR2737518, SRR2737519, SRR2737520, SRR2737521, SRR2737522, SRR2737523, SRR2737524, SRR2737525); Namibian cheetah “Rico” (GCA_003709585.1, SRR9855634, SRR9951918, SRR9951919, SRR9951920); and Tasmanian devil (GCA_902635505.1, ERS3900573). As the cheetah, wolverine, and Tasmanian devil samples had substantially higher sequence depth than the human samples, their alignment files were subsampled to ∼30× tiling using the “view” function of SAMtools to match approximately the human samples. Calls using ∼70× coverage before subsampling produced similar results as those using ∼30× coverage (data not shown). SNVs and indels were detected using the HaplotypeCaller function of GATK with parameter –minimum-mapping-quality 20 followed by the GATK function GenotypeGVCFs. The FILTER column of the VCFs was populated using the hard-filtering criteria suggested by the authors of GATK (https://gatk.broadinstitute.org/hc/en-us/articles/360035890471-Hard-filtering-germline-short-variants). For SNVs, these criteria were: QD < 2.0; MQ < 40.0; SOR > 3.0; FS > 60.0; MQRankSum < −12.5; and ReadPosRankSum < −8.0. For indels, these criteria were QD < 2.0; SOR > 10.0; FS > 200.0; and ReadPosRankSum < −20.0. Only high confidence variants after applying these filters were considered in the heterozygosity analysis. The degree of heterozygosity for each genome was tabulated based on the number of heterozygous variants per megabase of reference sequence.

Runs of homozygosity (ROH) were determined by counting the number of 25, 50, or 100 kb intervals in the genomes that did not contain any heterozygous SNVs (high confidence SNVs only; because of their higher error call rate and low incidence, indels were not considered for this analysis). For example, suppose that a given genome contained a heterozygous variant at position 200,000 of a given chromosome and another at position 550,000, with none in between. Then this would be counted as three runs [(550,000–200,000)/100,000] of homozygosity when using an interval size of 100 kb.

## Results and discussion

### Genome assembly: library construction, assembly strategy, assembly assessment, and annotation

Our male specimen of the North American wolverine for genome assembly was obtained from the Royal Ontario Museum tissue archive (sample no FN33715-3) (NCBI BioSample SAMN16725402). The specimen was originally deposited by the Northwest Territories Ministry of Natural Resources, sourced in 1990 from a licensed trapper from the Kugluktuk (Coppermine) region (67°48′43″N 115°05′05″W) of Nunavut in the Canadian Arctic (Fig. 1c). The sex and species identity of the sample were verified against the Barcode of Life Database and by the presence of the Y chromosome-specific gene, *SRY*.

Sizing of the purified wolverine DNA sample on the Agilent TapeStation showed a peak length of 18.9 kb with a trailing shoulder of longer DNA. From the TapeStation profile, we estimated only 10% of the DNA is >50 kb in length by mass (Fig. 2a). Normally, we would select genomic DNA samples with peak length >50–75 kb to make the optimal use of the CLR mode of the Pacific Biosciences’ (PacBio) Sequel II sequencer (Pacific Biosciences). Although the length of our wolverine DNA sample was less than optimal, DNA of this quality is acceptable for an archival tissue sample collected and stored ∼30 years ago. Since the sample has well-documented provenience and provenance, and tissue and DNA samples are available from the Royal Ontario Museum archive to other investigators, the sample proceeded to library construction and sequencing.

To improve the yield of long-reads from a suboptimal DNA sample, a post-library size-selection step was performed to deplete the shorter components of the library (see *Materials and methods*). Sequencing of the wolverine library was also increased from two to three PacBio Sequel II flow cells to achieve a greater representation of DNA at the long end of the size distribution. As a result, we achieved 96.9x coverage of the estimated size of the wolverine genome with reads having a read-length N50 of 16,453 bp. The longest CLR is 270,341 bp, with mean and median read-lengths of 13,236 and 12,445 bp, respectively. Most importantly, the dataset comprised significant coverage of the wolverine genome at the long end of the read distribution, as indicated by 2.6x genome coverage by reads having a N50 value greater than ∼60 kb (Fig. 2c). Hence, size selection of the library when combined with the use of an extra flow cell (three flow cells in total) could partially overcome the length deficiency of a suboptimal DNA sample. In total, 19,028,581 usable CLRs were generated, representing 251.8 Gb of sequences.

It is generally accepted that high-quality genome assemblies from large complex repetitive genomes is dependent on: (1) reads of sufficient lengths to span the repetitive regions of the genome; (2) sufficient and cost effective read coverage to sample the breadth and depth of the genome to enable the assembler to make confident calls during the contig-building process; and (3) the inherent accuracy of the reads, which affects both contig building and the base accuracy of the final assembly. At present, no sequencing platforms have arguably satisfied all three requirements for assembly of mammalian genomes. The CLR mode of the PacBio’s Sequel II sequencer perhaps comes the closest in providing maximal read lengths at a reasonable yield and cost, but falters by having reads with typical 15–20% pseudo-random errors in the forms of base substitutions and small insertions and deletions (Zhang *et al.* 2020). However, as shown by the present study, sequencing errors associated with CLR can be effectively mitigated.

Figure 3 is a schematic of our two-step assembly workflow for the wolverine genome. In Step-A, we used a strategy whereby long uncorrected PacBio CLRs are assembled by the Flye assembler (Kolmogorov *et al.* 2019) directly into a high-quality primary assembly, Gulo_gulo_luscus_F-V1.0. We favor this strategy over the computationally intensive approach of correcting individual PacBio reads prior to assembly (Koren *et al.* 2012), or the use of PacBio Circular Consensus Sequencing, also known as HiFi Sequencing (HiFi/CCS) (Lou *et al.* 2013; Wenger *et al.* 2019), where reads are corrected during sequencing. Currently, HiFi/CCS could only achieve a Q30 correction for only a relatively small portion of the templates in the flow cell, with the remaining templates producing a heterogeneous mixture of reads with varying degrees of correction. As it now stands, the productive yield of fully corrected sequences is too low for large genomes, requiring the inclusion of the partially corrected reads in order to achieve a desired genome coverage at an acceptable cost. Current computational models find assembling a mixture of reads of different accuracies daunting when compared with the established methodologies and computational models for assembling reads with a uniform, albeit high error rate. Moreover, the current HiFi/CCS process also has a marked detrimental effect on read-length, which could hinder the spanning of repetitive regions of a genome. Consequently, HiFi/CCS reads are presently better suited for making variant calls or for assembling smaller and less complex genomes with a more favorable cost to benefit calculation. With further development and cost reduction, HiFi/CCS could supersede other forms of single molecule sequencing. However, we opine that at this time, the use of longer and lower cost CLRs, albeit less accurate, is a better functional tradeoff for cost effective *de novo* assembly of large mammalian genomes. Here, we show the Flye assembler can efficiently assemble a high quality genome from uncorrected CLRs, and the ensuing polishing regimen could effectively mitigate sequence errors inherent to CLR sequencing.

In Step-B, we used Ragout 2 (Kolmogorov *et al.* 2018) to scaffold the primary assembly against the two available Mustelidae chromosomal-level assemblies, the ermine (*Mustela* *erminea*, mMusErm1.Pri) and the Eurasian river otter (*Lutra* *lutra*, mLutLut1.2), and a representative carnivore, the dingo (*Canis* *lupus dingo*, UNSW_AplineDingo_1.0). Ragout 2 infers evolutionary relationships between the submitted assembly against multiple reference genomes to build a presumptive scaffold based on the construction of hierarchical synteny blocks. However, any use of reference-assisted assembly or scaffolding should be done with caution. While the genomic architectures of phylogenetically related species are typically similar enough to provide useful long-range scaffolding information, nonetheless, the wolverine is neither an ermine nor a river otter, and there could be notable structural differences in the genomes. For instance, the three species have different diploid complements of 42, 44, and 36 chromosomes for the wolverine, ermine, and Eurasian river otter, respectively. Hence, over-aggressive fitting of an assembly onto reference genome(s) could give rise to assembly and scaffold artifacts. Artifacts are particularly acute for the shorter contigs where they are of insufficient lengths to demarcate and preserve true boundaries of syntenic blocks shared between the contigs and the reference genomes. As a general rule, a minimal contig N50 value of 1 Mb should be achieved before any assembly proceeds to reference-assisted scaffolding (Liu *et al.* 2018). However, even at this suggested minimal N50 value, a sizable portion of an assembled genome may still not be of sufficient length to avoid artifacts.

Reference-assisted scaffolding of our wolverine assembly was carried out conservatively. First, the contig N50 value for our primary assembly is 36.6 Mb, a value more than 30-fold higher than the minimal threshold suggested by Liu *et al.* (2018). We opine that a contig continuity of this magnitude would identify and, more importantly, preserve the vast majority of wolverine-specific syntenic blocks and their precise boundaries during the reference-assisted scaffolding process. Second, we only used reference genomes of related species that were assembled from long-reads and were scaffolded entirely from internal information such as those generated by Hi-C or optical mapping (Bionano Genomics, San Diego, CA), without unsupported inferences from other genomes. The reference genomes used here are such independent references. Third, we used Ragout 2, which accepts multiple reference genomes for scaffolding, with the rationale that taxonomic information from multiple reference genomes with different degrees of relatedness could improve accuracy by consensus agreement. The dingo (*C.* *lupus* *dingo*) assembly (UNSW_AlpineDingo_1.0) was included for this purpose, representing an outlier carnivore. However, we found the inclusion or exclusion of the dingo assembly did not materially affect the scaffold (data not shown). Finally, we were confident in the accuracy of our assembled contigs, and have disabled Ragout 2’s ability to split contigs to achieve better fit to the reference genomes during the scaffolding process. In cases where a contig in our wolverine assembly disagreed with one or more segments in the reference genomes, our contig was considered to be correct and wolverine-specific, and the contig was left intact and did not participate in the scaffolding process. Using the above criteria against the chromosomal-level reference assemblies of the ermine, Eurasian river otter, and dingo, 230 contigs in our primary assembly (Gulo_gulo_luscus_F-V1.0) participated in the scaffolding process and were organized by Ragout 2 into 19 scaffolds. The remaining 528 contigs in the primary assembly were left unplaced. The resulting presumptive chromosomal-level assembly for the wolverine after scaffolding was designated Gulo_gulo_luscus_R-V1.0 (WGS Accession: JAJHUB000000000).

Table 1 summarizes the assembly metrics of our short-read (Gulo_gulo_luscus_A-V1.0), long-read (Gulo_gulo_luscus_F-V1.0), and reference-assisted scaffolded assembly (Gulo_gulo_luscus_R-V1.0) for the wolverine. Immediately apparent is the marked improvement in assembly contiguity achieved by the use of long-reads. We observed nearly 2,400-fold improvement in the contig N50 and 1,000-fold improvement in the scaffold N50 statistics in our primary long-read assembly compared with an assembly derived solely from Illumina short-reads. Our primary assembly, Gulo_gulo_luscus_F-V1.0, achieved a contig N50 of 36.6 Mb, comprising 758 contigs and 705 scaffolds. The longest contig was 107 Mb, which alone represented nearly 4% of the genome. Eighty-eight contigs were organized by Flye into 35 scaffolds, resulting in a total scaffold count of 705 and a scaffold N50 of 50.8 Mb. Contig L50 and scaffold L50 were 20 and 14, respectively (i.e. 50% of the genome is represented by the 20 and 14 longest contigs and scaffolds, respectively). Following reference-assisted scaffolding using the Eurasian river otter, ermine, and the dingo genomes, the resulting Gulo_gulo_luscus_R-V1.0 assembly saw a further improvement in scaffold statistics with a final scaffold N50 of 144.0 Mb and a scaffold L50 value of 7.

**Table 1.** Assembly metrics. Comparison of North American wolverine assemblies with other Mustelidea.

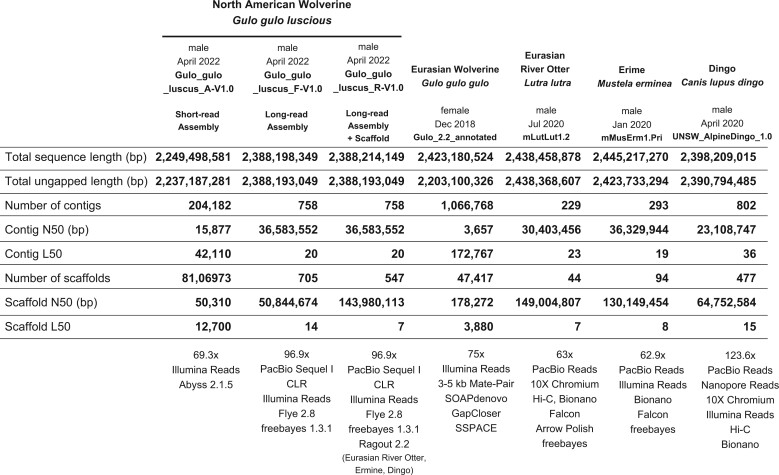

We then compared our assemblies with those previously reported for Mustelidae. As indicated by the contig N50 metrics in Table 1, our short-read assembly (Gulo_gulo_luscus_A-V1.0) is more continuous than the short-read assembly previously reported for the Eurasian wolverine (Ekblom *et al.* 2018; Gulo_2.2_annotated), although the latter assembly has better scaffold statistics, presumably due to the author’s use of mate-pair reads. Our long-read assembly (Gulo_gulo_luscus_R-V1.0) is *at par* with or has better contiguity than the reported chromosomal-level reference assemblies for the ermine (mMusErm1.Pri), Eurasian river otter (mLutLut1.2), and the outgroup control, the dingo (UNSW_AlpineDingo_1.0).

### Contamination in the assembly

Since the original wolverine tissues were collected in the field, we looked for potential microbial and other environmental DNA contaminants in our assembly. As PacBio CLRs have 15–20% pseudo-random errors in the forms of base substitutions and small insertions and deletions (Zhang *et al.* 2020), a contaminant search at the read level before the assembly and error correction steps would not be sensitive, and may have unacceptable false positive or false negative hits. While Illumina short-reads are accurate, they could be too short to make reliable and unambiguous calls. Consequently, we directed the contamination assessment step to the final assembly. Analysis was done in 5 kb windows (see *Materials and methods*). Fifty-eight of the 478,025 windows (0.01%) showed no significant BLAST hits, presumably representing unique wolverine sequences. Analysis of the remaining 477,967 windows revealed no microbial or fungal sequences amongst the top 50th ranked hits, indicating there were no overt environmental contaminants in the Gulo_gulo_luscus_F-V1.0 assembly.

To assess the overall composition of the assembly, we tabulated the results from BLAST analysis against a series of sequential filters of decreasing phylogenetic distance from the wolverine. The results of this tabulation are presented in Supplementary Fig. 1. As expected, a majority of windows (475,532 windows; 99.27%) could be assigned with high confidence to contigs in the ermine (GCF_009829155.1) or the Eurasian river otter (GCA_902655055.2) assemblies, with an additional 961 windows (0.20%) to the genomes of other members of the Mustelidae, including the ferret, mink, or marten. Of the remaining 2,381 non-Mustelidae windows, 1,849 (0.39% of total) had plausible hits to other members of the carnivora lineage, in which 1,718 (0.36%) and 131 (0.03%) hit unannotated sequences in the dog and cat genomes, respectively. Five hundred and thirty-two windows (0.05%) hit a variety of other carnivore species, including the brown bear, seal, and panda. The remaining 93 windows hit taxonomic lineages outside carnivora; notably, 86 (0.02%) to diverse mammalian species, with two to plant and fish, and one to virus. We believe that most if not all of the non-Mustelidae hits are fortuitous. In all cases, the BLAST hit regions are piecemeal, of low alignment identity, and do not extend to adjacent or to any other 5 kb windows in the assembly. Finally, we also looked for potential laboratory contaminants. Abundant human DNA contamination has been reported in many nonprimate genome databases (Longo *et al.* 2011). Presumably, these sequences arose through cross-contamination between samples within the sequencing facilities that routinely process human samples, or from contaminations as a result of sample handling by humans. Using a primate specific SINE, *AluY* probe (Longo *et al.* 2011), we found no primate sequences in the Gulo_gulo_luscus_F-V1.0 assembly.

### North American wolverine mitochondrial genome

We report the first full-length mitochondrial genome assembled for the North American wolverine (Gulo_gulo-luscus_F-V1.0; contig WOV01_MT20201101) (Supplementary Fig. 2). The accuracy of our mitochondrial assembly is supported by uninterrupted mapped Illumina reads tiled across the length of the assembly in 50 bp moving windows of single base increments. Tiling depths of greater than 10,000 reads were tabulated across 95% of the assembled mitochondrion genome (Supplementary Fig. 2b). The remaining 5% of the mitochondrial genome, notably in the D-loop region, is more difficult to map with short-reads due to the presence of strings of short repetitive sequences. Although tiling depth was lower in the D-loop region, it did not fall below 4,000 reads. Importantly, the identical mitochondrial genome of 16,556 bp was independently assembled from both uncorrected long-reads (having 15–20% sequence error), and from highly accurate Illumina short-reads (sequence accuracy >99.9%, >Q35).

A phylogenetic neighbor-joining tree based on the mitochondrial genomes of selected members of the Mustelidae is presented in Supplementary Fig. 2c. Within sampling limitations, the North American wolverine is clustered with, but is distinct from the three mitochondrial genomes of the Eurasian wolverine reported in GenBank, NC_009685.1, KF415127.1, and KR611313.1. The latter specimen, KR611313.1, is from the Great Khingan Mountains of China. Wolverines from Northeast China are reported to have a generally smaller body plan and baculum morphology compared with the other Eurasian wolverines (Zhu *et al.* 2016). The constructed mitochondrion tree reflects the dichotomy between wolverines from Scandinavia and Northeast China.

BLAST analysis of the wolverine mitochondrial genome against the Gulo_gulo_luscus_F-V1.0 assembly at a threshold of 1E−4 revealed 200 potential mitochondrial pseudogene loci of varying lengths and degeneracies (data not shown). These so termed “NUMTs” (Nuclear Mitochondrial DNA) are believed to originate from invasion of the nuclear genome by mitochondrial DNA via nonhomologous recombination (Richly and Leister 2004). It is generally viewed that the accumulation of NUMTs is a continuous evolutionary process (Triant and DeWoody 2007). Using the same threshold, the mouse genome (*Mus musculus*) has 190 copies, the rat genome (*Rattus norvegicus*) has 61 copies, and human genome has 1,356 copies (Richly and Leister 2004).

### Repetitive sequences

Repeat elements described in RepBase were tabulated in our Gulo_gulo_luscus_F-V1.0 assembly using RepeatMasker (Tarailo and Chen 2009). Table 2 summarizes the distributions of repetitive sequences in the wolverine genome. Matches covered 32.4% of the genome, including 2.8% SINEs, 19.5% LINEs, 4.3% LTR elements, and 2.1% simple repeats, with the remainder comprising other repeat classes.

**Table 2.** Repetitive sequences in the wolverine genome.

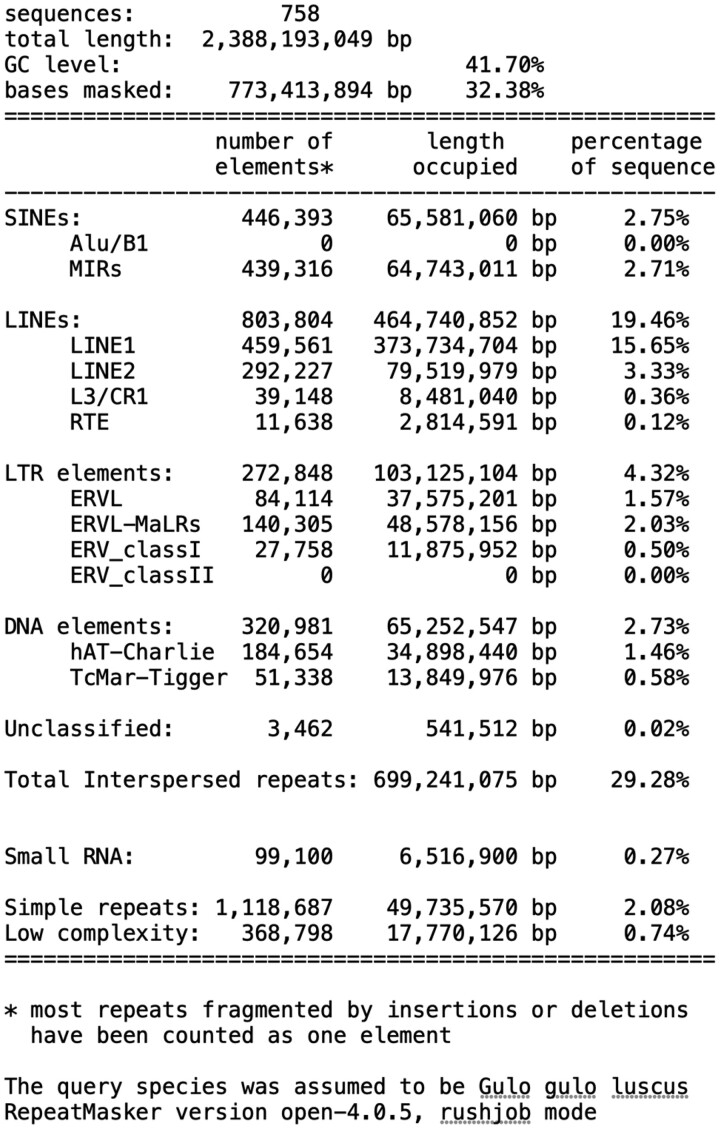

### Microsatellite markers

Microsatellite markers have been developed to assess gene flow, reproductive success, and population diversity in mustelid species (Supplementary Table 1 and references therein). Only a small number of microsatellite markers have been developed specifically for the wolverine, and have instead been developed for the other mustelid species such as marten, badger, otter, or mink. Although some markers developed for these species have been reported to cross-react with the wolverine, they have not been systematically tested. Supplementary Table 1 is a tabulation of the 169 reported mustelid microsatellite loci and their status in the North American wolverine genome in a searchable and sortable format. Of the 23 loci previously developed and reported for the wolverine, all are predicted to produce informative products from our specimen when amplified with the appropriate pairs of primers. Allowing up to three single-base mismatches in one or both PCR primers, our analysis showed that 88 (60.3%) of 146 microsatellite markers developed for the other mustelids are potentially informative for the North American wolverine. In the event investigators wish to have more efficient amplification from these primers, Supplementary Table 1 also provides a revised primer design where mismatches to the wolverine genome have been adjusted. It should be feasible for the community to mine the present assembly to develop additional new microsatellite markers for the wolverine using informatics search algorithms.

It should be noted that among the small number of microsatellite markers examined to date, they have not been found to be particularly polymorphic in the wolverine (Walker *et al.* 2001; Väli *et al.* 2008). As a consequence, Ekblom *et al.* (2021) have moved to the use of SNV genotyping panels in conservation monitoring of the Scandinavian wolverine population. If this deficiency proved to be a general property of the wolverine microsatellites, a similar strategy that does not rely upon microsatellite markers might need to be adopted for the North American wolverine.

### BUSCO analysis and exon-level gene annotation

The BUSCO (Benchmarking Universal Single-Copy Orthologs) program has long been used to assess the completeness of genome assemblies (Simão *et al.* 2015). When BUSCO 5.2.2 and its 9,226 genes of the Mammalia gene group was tested on our wolverine Gulo_gulo_luscus_F-V1.0 assembly, 8,936 genes (96.86%) were scored as complete, 67 genes (0.73%) were scored as fragmented, and 223 genes (2.42%) were scored as missing in the assembly. At face value, these BUSCO scores would place our wolverine assembly at the top echelon for reported genome assemblies derived from long-reads.

**Fig. 4.**
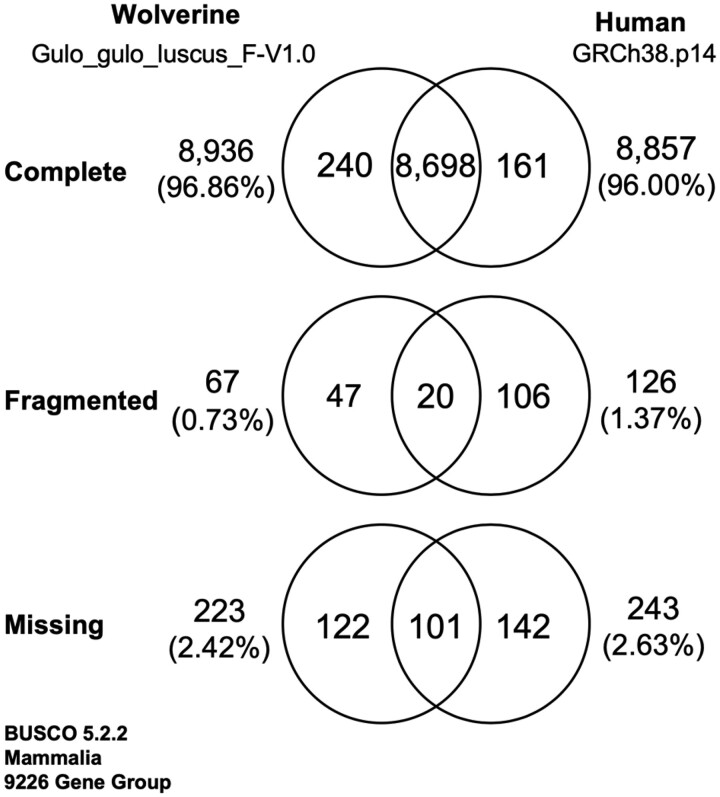
BUSCO 5.2.2 results (Benchmarking Universal Single Copy Orthologs; mammalia_odb10) (Simão *et al.* 2015). Venn diagram comparison of wolverine assembly (Gulo_gulo_luscus_F-V1.0) with human GRCh38.p14 (see *Materials and methods*).

A defining feature of BUSCO is its ability to assess the quality of genome assemblies across a broad range of species. This convenient feature was accomplished by balancing sensitivity and specificity to create a single universal set of unbiased consensus gene profiles that could be used generically. As a consequence that gene profiles are not optimized to any particular species, subsets of them could have difficulties detecting their cognate genes in species whose orthologs deviate significantly from the consensus. In this way, BUSCO could under-count those genes, resulting in an underestimation of the quality of the assembly under test. This concern was bored out when we compared BUSCO results for the wolverine assembly with that of the gold-standard reference genome, the current build of the human reference genome, GRCh38.p14 (2022 February 3) (GCF_000001405.40). In view of the recognized completeness of the official human reference genome, we expected a near-perfect BUSCO score for GRCh38.p14. Instead, BUSCO scored GRCh38.p14 similarly and perhaps marginally less well when compared with our wolverine assembly, with 8,857 (96.00%) genes as complete, 126 genes (1.37%) as fragmented, and 243 genes (2.63%) as missing (Fig. 4). Examination of the NCBI Genome Data Viewer revealed all 369 genes that BUSCO had failed, were, in fact, present and experimentally verified as complete in GRCh38.p14. Thus, the problem appears to lie with BUSCO and not deficiencies in the human reference genome. With this finding, we examined the 290 genes BUSCO had designated as missing or incomplete in our wolverine assembly. Using the exon-level annotation method described in the *Materials and methods*, 288 of the 290 genes were found to be present and complete, revising the BUSCO completion score from 96.86% to 99.98% for the wolverine assembly. A list of those 288 genes and their Accessions is presented in Supplementary Table 2, with their exon coordinates annotated in the assembly.

Only two BUSCO genes could not be found in the wolverine assembly, intraflagellar transport 172 (*IFT172*; BUSCO group 6011at40674), and solute carrier family 10 member 5 (*SLC10A5*; BUSCO group 166011at40674). *IFT172* is a single copy gene comprising 49 exons in human. Wolverine and the ermine appear to have multiple closely linked *IFT172*-like genes interspersed with highly repetitive sequences that might have prevented their assembly in both species (data not shown). Human and rodent *SLC10A5* is an intron-less gene in a family of seven solute transporters. Using the approach that was successful for annotating nearly all of the BUSCO genes (see *Materials and methods*), we could not identify *SLC10A5* in our wolverine assembly. The search included a 14 kb region between *ZFAND1* and *IMPA1* on contig WOV01_AAE20220223_F8-ctg00009 where we expected to find wolverine *SLC10A5* based on synteny with human, mouse, ermine, and wolverine genomes. A sensitive BLAST search of this region revealed the presence of extensive fragmented *SLC10A5*-like sequences with multiple frame-breaking insertions and deletions, indicative of a pseudogene (data not shown). Although we cannot rule out the existence of a functional *SLC10A5* elsewhere in the wolverine genome, during the course of annotating the other six members of the wolverine *SLC10* family [*SLC10A1* (OM350569); *SLC10A2* (OM350570); *SLC10A*3 (OM350571); *SLC10A4* (OM350572); *SCL10A6* (OM350573); and *SLC10A*7 (OM350574)], we did not encounter any other *SLC10*-like sequences that could be a *SLC10A5* candidate in the wolverine. Since it is still formally possible that *SLC10A5* might exist in wolverine on a DNA segment that is not in our assembly, we therefore conservatively scored this gene as the second negative gene in our BUSCO tabulation.

### Wolverine *TTN*, *DMD*, and *CNTNAP2* genes for assessment of assembly quality

**Fig. 5.**
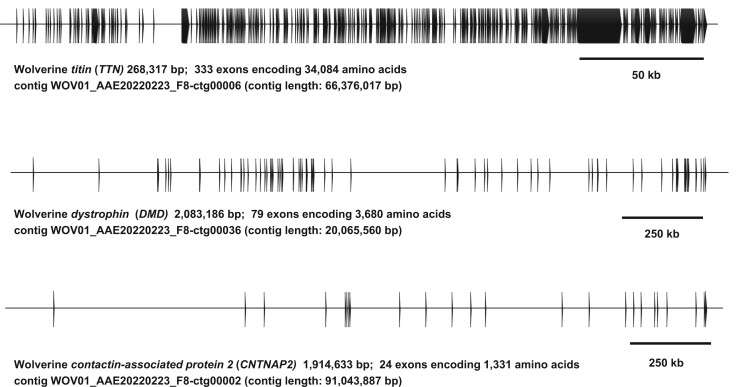
Wolverine titin (*TTN*) (OM350600), dystrophin (*DMD*) (OM350512), and contactin-associated protein 2 (*CNTNAP2*) (OM350695). The long-range contiguity of the wolverine assembly is exemplified by these three benchmarked genes noted for their size or the large number of coding exons. Predicted exons are denoted by boxes with the direction of transcription from left to right. Accessions for the predicted wolverine orthologs are provided in parentheses.

We used three other benchmark genes to spot-check the continuity and quality of our assembly. Two genes, dystrophin (*DMD*) and contactin-associated protein 2 (*CNTNAP2*), are among the longest known mammalian genes with exons spanning more than a million bases depending on the species, and provide a useful metric to assess the long-range contiguity of our assembly. As shown in Fig. 5, *DMD* (OM350512), and *CNTNAP2* (OM350695) are complete in the wolverine assembly and are situated on contigs of 20 and 91 Mb lengths, respectively. The 79 exons of *DMD* spanned 2,083,186 bp, and the 24 exons of *CNTNAP2* spanned 1,914,633 bp. All assigned exons are flanked by the expected consensus acceptor and donor splice sites and are in the correct order when compared with the exons of the human and mouse orthologues.

The third benchmark gene is titin (*TTN*). In contrast to *DMD* and *CNTNAP2*, *TTN* spans only 268,317 bp, but *TTN* is generally acknowledged to have the highest exon count amongst mammalian genes and encodes the largest polypeptide. Wolverine *TTN* comprises at least 333 exons and encodes a massive polypeptide of 34,084 amino acids (OM350600) (Fig. 5). We exploited the high exon count and the long contiguous open reading frame (ORF) of wolverine *TTN* to estimate the level of residual CLR sequencing errors that have escaped the polishing steps in the final assembly. In the absence of sequencing errors, *TTN* should encode an ORF of 34,084 amino acids with a single in-frame termination codon at the end. Of the 64 possible translation codons, 18 codon species could create an in-frame termination codon from a single base change. These codon species in wolverine *TTN* thereby provide 12,075 nucleotide positions where single base sequencing errors could easily be discernible. When these positions are combined with the canonical acceptor (AG) and donor (GT) splicing signals flanking each exon, the total nucleotide positions from which we can unambiguously discern a single base sequencing error in wolverine *TTN* is 13,403. Since we did not observe any interruptions to the *TTN* ORF or deviations from the consensus splicing signals for any of the 333 exons, we can estimate an upper limit for residual single base sequencing errors in our assembly to be no greater than 1 in 13,403 (0.007%). When the ORFs encoded by the 79 exons of *DMD* and the 24 exons of *CNTNAP*2 are similarly tabulated and combined with the result from *TTN*, we can revise the estimated upper limit for residual single base substitution errors to be no greater than 1 in 15,777 (0.006%), which corresponds to a Phred quality score >40. This value is likely to be an underestimation, since we did not detect disruptions of exons amongst the 897 genes manually curated in the present study and for the genes annotated as described in the *Materials and methods* (see Supplementary Tables 2 and 3).

### Annotation of selected wolverine genes: wolverine *SRY*

A male specimen is typically selected for mammalian genome assembly in order to include the Y chromosome. The sex of our wolverine specimen was confirmed as a male by the presence of the Y chromosome-specific gene, *SRY* (Sex-determining Region Y) in the assembly. Wolverine *SRY* (OM569651) is an intron-less gene (encoding 291 amino acids) having 92.7% sequence identity with Eurasian river otter *SRY* (AB491588.1), 94.5% sequence identify with ermine *SRY* (XM_032331906.1), and 61.4% sequence identity with human *SRY* (NM_003140.3). Interestingly, there are two copies of *SRY* in wolverine. The two genes are separated by 3 kb and transcribed in divergent directions. The second gene, “*SRY-Like” (SRYL)* (OM569650), is nearly but not identical to *SRY*, encoding a 23 amino acid long N-terminal extension that is not found in the first gene. The ermine also appears to have two *SRY* sequences, *LOC116583904* and *LOC116583905*, separated by a similar distance and transcribed divergently as in the wolverine. In contrast, the two ermine genes encode an identical polypeptide and without the N-terminal extension found in wolverine *SRYL*. *SRY* is typically a single copy gene in most mammals, but multiple copies are found in old world rodents (Nagamine 1994).

### Genes associated with aggressive traits and behavior

Despite there being no recorded cases of attacks on people, their avoidance of areas with human activity, and being seldom seen due to their low population density in remote habitats, wolverines have nevertheless maintained a negative perception among the public (Bonamy *et al.* 2020 and references therein). Deserving or not, their alleged ferocity and tenacity have been personified by a multitude of sports teams engaged in contact sports, and are further reinforced by the popular media. Truth be told, a wide range of cultural, social, economic, and psychological factors influence relationships with the wolverine (review Bonamy *et al.* 2020). In many Indigenous societies, the wolverine is a cultural keystone species and is viewed as a trickster. In a recent survey (Bonamy *et al.* 2020), Dene and Métis trappers and hunters in the Canadian Northwest Territories recognized the wolverine’s intelligence and valued their pelts, but were annoyed by the theft of baits and the raiding of live traps. Contrary to popular perception, none of the respondents were fearful of wolverines even in relatively close proximity. That said, aggressive behaviors are believed to drive competition for food, mates, and other adaptions to a harsh, resource limiting environment (Anholt and Mackay 2012). To these ends, the wolverine is apparently well adapted, as shown by their dominance and demeanor at carrion sites (Klauder *et al.* 2021). From genetic and evolutionary perspectives, aggressive behavior at the individual or at the species level could be viewed as a heritable quantitative trait attributable to multiple segregating genes that are influenced by the environment and are under stabilizing selection (Falconer and Mackay 1996; see also reviews Veroude *et al.* 2015; Fernandez-Castillo and Cormand 2016). As a resource to study the interplay of genes with the environment in the wolverine, we provide a list of annotated wolverine genes whose orthologues have been shown to be associated with aggressive dominance traits in human and animal models, association studies, and pathway analysis (Supplementary Table 2, and references therein).

Alterations in gene dosage through copy number variation have been shown to be a mechanism for mediating phenotypic traits (Schrider and Hahn 2010). For example, reduced cancer incidence in large long-lived animals such as elephants, could be due to increased copy number of the tumor suppressor gen*e TP53* (Abegglen *et al.* 2015). Amylase gene copy numbers have also been shown to correlate with increased starch consumption in different populations (Pajic *et al.* 2019). The behavior associated genes depicted in Supplementary Table 2 appear to be present in single copy in the wolverine haploid genome based on the assembly and read depth analysis of mapped Illumina reads (data not shown). This finding suggests that behavior in wolverine, at least with respect to these genes, is not mediated by a simple gene dosage mechanism. Like many complex traits with environmental components, behavioral traits likely involve the interplay of multiple genomic, epistatic, and epigenomic determinants. The present assembly and annotation provide a resource for such investigations.

### Genes of the innate immunity pathways

There has been a marked increase in outbreaks of infectious disease among wildlife attributable to environmental disruptions from anthropogenic climate changes (reviews Omazic *et al.* 2019; Cohen *et al.* 2020). Arctic species are particularly vulnerable as pathogens invade new northern niches. The innate immune system is the first to respond to pathogens (review Paludan *et al.* 2021). As a resource to support infection genomics and wildlife management, we provide gene annotations for the principal members of the innate immunity pathways in the wolverine (Table 3 and Supplementary Table 2).

**Table 3.** Selective principal members of the innate immunity pathways in wolverine: the pattern recognition receptors, interferons, and intermediaries.

**GENE SYMBOL**	**ALTERNATIVE NAME/ SYMBOL**	**ACCESSION**

**Toll-Like Receptors**	* *	
***TLR1***	*CD281*	OM291787
***TLR2***	*CD282*	OM291788
***TLR3***	*CD283*	OM291789
***TLR4***	*CD294*	OM291790
***TLR5***	*CD295*	OM291791
***TLR6***	*CD296*	OM291792
***TLR7***	*CD287*	OM291793
***TLR8***	*CD288*	OM291794
***TLR9***	*CD289*	OM291795
***TLR10***	*CD290*	OM291796
** **	** **	** **
**Nod-Like Receptors**	**NLR family pyrin domain containing**	
***NLRP1***	*NALP1; CARD7*	OM291738
***NLRP3***	*NALP3*	OM914421
***NLRP5***	*NALP5*	OM291739
***NLRP6***	*NALP6*	OM291740
***NLRP8***	*NOD16*	OM291741
***NLRP10***	*NOD8; NALP10*	OM291742
***NLRP12***	*NALP12*	OM291743
***NLRP13***	*NOD14; NALP13*	OM291744
***NLRX1***	*NOD5*	OM914422
***NOD1***	*NLRC1; CARD4*	OM291746
***NOD2***	*NLRC2; CARD15*	OM291747
***NLRC3***	*NOD3*	OM291736
***NLRC5***	*NOD4*	OM291737
** **	** **	** **
**C-Type Receptors**	**C-type lectin domain family**	
***CD209***	*CLEC4L; DC-SIGN*	OM291570
***OLR1***	*CLEC8A*	OM291748
***CLEC1A***	*CLEC1*	OM914415
***CLEC1B***	*CLEC2; CLEC2B*	OM291571
***CLEC4D***	*CD368; CLEC6; CLECSF8; DECTIN-3*	OM291572
***CLEC4E***	*CLECSF9; MINCLE*	OM291573
***CLEC4G***	*DTTR431*	OM291574
***CLEC5A***	*CLECSF5*	OM291575
***CLEC7A***	*CLECSF12; DECTIN-1*	OM291576
***CLEC9A***	*CD370; DNGR-1*	OM291577
***CLEC12A***	*CD371*	OM291578
** **	** **	** **
**Rig-Like Receptors**	**Retinoic acid-inducible gene-l-like receptors**	
***DDX58***	*RIGI; DExD/H-box helicase 58; RLR1*	OM291581
***DHX58***	*RLR-3; LPG2; RLR3*	OM569636
***MAVS***	*CARDIF; IPS-1; IPS1; VISA*	OM291730
***IFIH1***	*MDA5; IDDM19; RLR; 2IDDM19*	OM291621
***ZBP1***	*Z-DNA binding protein 1*	OM291816
** **	** **	** **
**Adaptors**		
***MYD88***	*IMD68; MYD88D*	OM291731
***STING1***	*ERIS*	OM291777
***TICAM1***	*TRIF; MyD88-3*	OM569652
***TIRAP***	*MYD88-2*	OM291786
***UNC93B1***	*UNC93; TLR signaling regulator*	OM569655
***TRAF1***	*TNF receptor associated factor 1*	OM291807
***TRAF2***	*TNF receptor associated factor 2*	OM291808
***TRAF3***	*TNF receptor associated factor 3*	OM291809
***TRAF4***	*TNF receptor associated factor 4*	OM291810
***TRAF5***	*TNF receptor associated factor 5*	OM291811
***TRAF6***	*TNF receptor associated factor 6*	OM291812
***TRAF7***	*TNF receptor associated factor 7*	OM291813
**Janus And Other Kinases**	** **	
***JAK1***	*Janus kinase 1*	OM291672
***JAK2***	*Janus kinase 2*	OM291673
***JAK3***	*Janus kinase 3*	OM291674
***TYK2***	*Janus kinase JTK1*	OM291814
***IRAK1BP1***	*AIP70; SIMPL*	OM291655
***IRAK1***	*IRAK-1*	OM291656
***IRAK2***	*IRAK-2*	OM794762
***IRAK3***	*IRAKM*	OM291657
***IRAK4***	*IRAK-4*	OM291658
***RIPK1***	*RIP-1*	OL505550
***RIPK2***	*RIP-2; CARD3*	OM291755
*** ***	*** ***	*** ***
**STATs**	** **	
***STAT1***	*Signal transducer/activation of transcription 1*	OM291770
***STAT2***	*Signal transducer/activation of transcription 2*	OM291771
***STAT3***	*Signal transducer/activation of transcription 3*	OM291772
***STAT4***	*Signal transducer/activation of transcription 4*	OM291773
***STAT5A***	*Signal transducer/activation of transcription 5A*	OM291774
***STAT5B***	*Signal transducer/activation of transcription 5B*	OM291775
***STAT6***	*Signal transducer/activation of transcription 6*	OM291776
*** ***	*** ***	*** ***
**Interferon Pathway**		
***IFNA1***	*IFN-alpha 1*	OM350528
***IFNA2***	*IFN-alpha 2*	OM350529
***IFNA3***	*IFN-alpha 3*	OM350530
***IFNA4***	*IFN-alpha 4*	OM350531
***IFNA5***	*IFN-alpha 5*	OM350532
***IFNA6***	*IFN-alpha 6*	OM350533
***IFNA7***	*IFN-alpha 7*	OM350534
***IFNA8***	*IFN-alpha 8*	OM350535
***IFNA9***	*IFN-alpha 9*	OM350536
***IFNA10***	*IFN-alpha 10*	OM350537
***IFNA11***	*IFN-alpha 11*	OM350538
***IFNB1***	*IFN-beta 1*	OM291625
***IFNE***	*IFN-epsilon*	OM350539
***IFNG***	*IFN-gamma*	OM291628
***IFNK***	*IFN-kappa*	OM291629
***IFNL1***	*IFN-lambda 1; IL-29*	OM291630
***IFNL2***	*IFN-lambda 2a; IL-28A*	OM291631
***LTA***	*Interferon B*	OM291678
***IFNAR1***	*Interferon-alpha R1*	OM291623
***IFNAR2***	*Interferon-alpha R2*	OM291624
***IFNGR1***	*CD119; IFN-gamma R1*	OM291626
***IFNGR2***	*IFN-gamma R2*	OM291627
***IFNLR1***	*IFN-lambda R1; IL-28 R1; IL-28 RA*	OM291632
***IL10RB***	*CD210B; CRFB4; IL-10R2; IL-10 RB; IL-28R*	OM291646
***IRF1***	*Interferon regulatory factor 1*	OM291659
***IRF2BP1***	*Interferon regulatory factor 2 BP1*	OM291660
***IRF2BP2***	*Interferon regulatory factor 2 BP2*	OM291661
***IRF2BPL***	*Interferon regulatory factor 2 BPL*	OM291662
***IRF2***	*Interferon regulatory factor 2*	OM291663
***IRF3***	*Interferon regulatory factor 3*	OM291664
***IRF4***	*Interferon regulatory factor 4*	OM291665
***IRF5***	*Interferon regulatory factor 5*	OM291666
***IRF6***	*Interferon regulatory factor 6*	OM291667
***IRF7***	*Interferon regulatory factor 7*	OM291668
***IRF8***	*Interferon regulatory factor 8*	OM291669
***IRF9***	*Interferon regulatory factor 9*	OM291670
***MAVS***	*IPS1; VISA; CARDIF*	OM291730
***IFRD1***	*Interferon dev regulator 1*	OM291633

See Supplementary Table 2 for wolverine orthologs for the mitogen-activated protein kinases (MAPKs), beta-defensins, interleukins, tumor necrosis factors, and the chemokines under their respective tabs.

Table 3 highlights the major classes of cell surface and intracellular innate receptors of innate immunity system, and their principal signaling intermediaries in the wolverine. These receptors include the Toll-like (Kawai and Akira 2011), Nod-like (Franchi *et al.* 2009), C-type lectin (Bermejo-Jambrina *et al.* 2018), and the RIG-I (Rehwinkel and Gack 2020) families of receptors that recognize pathogen-associated patterns or damage-associated molecular patterns. DHX58 (RIGI) and IFIH1 (MDA5) are the principal sensors for nucleic acids from viral pathogens (Kawai and Akira 2006). Once receptors are activated, signals are potentiated into the cell nucleus by specific adaptor molecules such as MYD88, TICAM1 (TRIF), MAVS, and STING (Chen and Jiang 2013), and a series of signaling intermediaries, notably the STATs and members of the IRAK, JAK (Bousoik and Alibadi 2018) and the mitogen-activated protein (MAPK) families of kinases (Arthur and Ley 2013). Signals potentiated through MAPKs could also provide cross-talk with the adaptive immunity pathways, and the support of apoptosis or autophagy leading to the removal of damage or infected cells. The 55 *MAPK* genes in wolverine are provided in Supplementary Table 2 under the *MAPK* tab.

Host responses upon receptor activation include the expression of interferons (McNab *et al.* 2015) (Table 3), specific inflammatory cytokines (review Abraha 2020), chemokines (review Esche *et al.* 2005), and antimicrobial peptides such as the defensins (Xu and Lu 2020). Chemokines, notably CXCL7, CXCL9-11, CCL20, and CCL28, appear to have direct antimicrobial activities similar to the defensins (Esche *et al.* 2005). We identified 26 beta defensin genes in wolverine (Supplementary Table 2). All paralogs of the large extended human interleukin-17 (*IL17A* to *IL17F*) and interleukin-17 receptor (*IL17RA* to *IL17RE*; *IL17REL*) families are present in wolverine, pointing to a broad evolutionary conservation of all individual family members in the rapid response to infectious agents (review Valeri and Raffatellu 2016). The chemokines, interleukins, and tumor necrosis factors and receptors involved in wolverine innate immunity are presented in Supplementary Table 2.

**Fig. 6.**
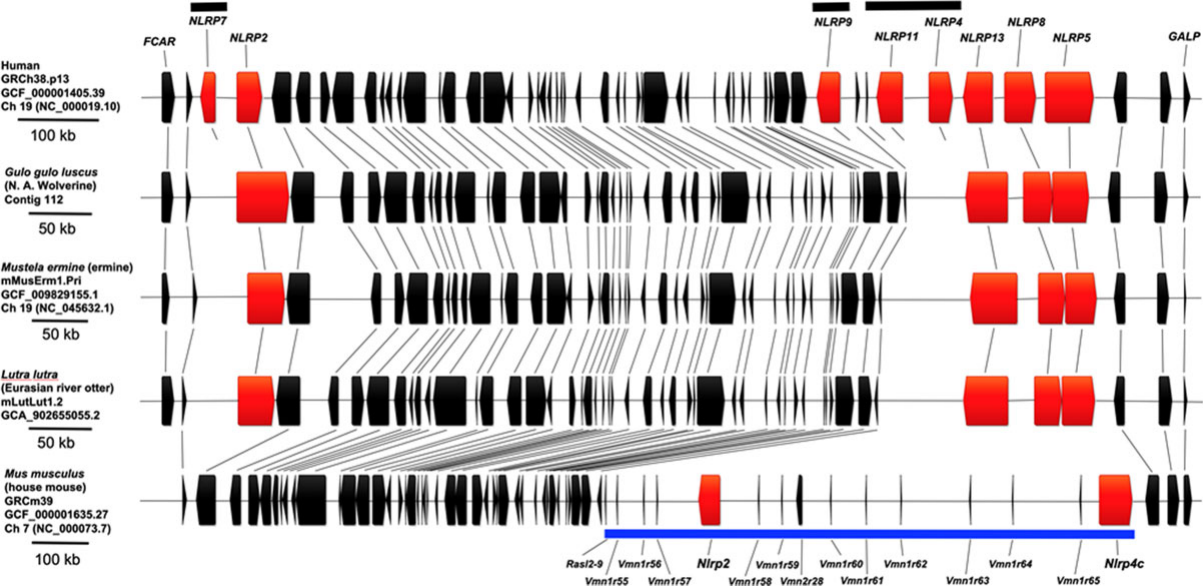
Human chromosome 19 region spanning *FCAR* to *GALP* aligned to the corresponding syntenic regions of the wolverine, ermine, European river otter, and house mouse genomes. Boxes denote the annotated coding exons and intervening introns for each gene. The direction of transcription is indicated by the outdent on the left or the right edge of the box. Human, ermine, and mouse gene boundary coordinates were downloaded from the respective online genome browsers. Gene boundaries for the Eurasian river otter gene were manually annotated from the assembly, mLutLut1.2. For illustrative purpose, DNA segments for the wolverine, ermine, Eurasian river otter, and mouse are normalized to the length of the DNA spanning human *FCAR* and *GALP*. Scaling factors for this normalization process are denoted by the scale bars for each respective species. Orthologous genes in the different species are indicated by thin lines. Gene order from left to right on human chromosome 19 is as follow: *FCAR* (OM291614); *NCR1* (OM291733); *NLRP7* (−); *NLRP2* (OM569642); *GP6* (OM291619); *RDH13* (OM291754); *EPS8L1* (OM291611); *PPP1R12C* (OM291750); *TNNT1* (OM291805); *TNNI3* (OM291804); *DNAAF3* (OM291610); *SYT5* (OM291778); *PTPRH* (OM291753); *TMEM86B* (OM291797); *PPP6R1* (OM291751); *HSPBP1* (OM291620); *BRSKI* (OM291567); *TMEM150B* (OM291798); *KMT5C* (OM291677); *COX6B2* (OM291579); *GARIN5B* (OM291618); *IL11* (OM291648); *TMEM190* (OM291799); *TMEM238* (OM291800); *RPL28* (OM291756); *UBE2S* (OM291815); *SHISA7* (OM291768); *ISOC2* (OM291671); *C19orf85* (OM291568); *ZNF628* (OM291821); *NAT14* (OM291732); *SSC5D* (OM291769); *SBK2* (OM291766); *SBK3* (OM291767); *ZNF579* (OM291818); *FIZ1* (OM291616); *ZNF524* (OM291817); *ZNF865* (OM291823); *ZNF78*4 (OM291822); *ZNF580* (OM291819); *ZNF581* (OM291820); *CCDC106* (OM350495); *U2AF2* (OM350601); *EPN1* (OM350513); *NLRP9* (−), *RFPL4A* (OM350565); *RFPL4AL1* (−); *NLRP11* (−); *NLRP4* (−); *NLRP13* (OM291744); *NLRP8* (OM291741); *NLRP5* (OM291739); *ZNF787* (OM350609); *ZNF444* (OM350608); and *GALP* (OM291617). (−) denotes gene ortholog not found in the present wolverine assembly. Members of the Nod-like receptor family are represented in red. Black bar indicates human genes not present in the wolverine, ermine, and Eurasian river otter alignment. Blue bar indicates a mouse gene segment that is not syntenic to human and the other species. Accessions for predicted wolverine orthologs are provided in parentheses.

We were able to identify and annotate the vast majority of the presumptive wolverine orthologs of genes described for the human innate immunity pathways, with the notable exceptions of *NLRP4, NLRP7*, *NLRP9, CLEC4A, CLEC4C*, and *CLEC4M (CD299).*Figure 6 shows a segment of human chromosome 19 containing eight Nod-like receptor genes. The region from *FCAR* to *GALP* is aligned with the corresponding syntenic regions on the wolverine, ermine, European river otter, and the house mouse genomes (mouse *Fcar* is not syntenic). Notably, synteny in this region in the mouse is only partial as indicated by an insertion of a DNA segment containing vomeronasal (pheromone) receptor genes and the Nod-like receptor genes, *Nlrp2* and *Nlrp4c. NLRP11* is known to be primate-specific and is not expected to be found in the other species. Despite the overall conservation of gene order and gene orientation in all five species, Nod-like receptor genes, *NLRP4, NLRP7*, and *NLRP9* are absent in the wolverine at their expected syntenic locations. Tiling of long- and short-reads across these regions of the wolverine contig showed no evidence for localized mis-assembly that could explain the absence of these genes. A BLAST search also failed to identify these genes elsewhere in our wolverine assembly. Likewise, *NLRP4, NLRP7*, and *NLRP9* are also absent in the ermine and European river otter assemblies. Human *NLRP4* is reported to be a negative regulator for autophagy and interferon signaling, and *NLRP7* and *NLRP9* are implicated in the inflammasome regulation and reproduction (review Carriere *et al*. 2021). Since many pattern recognition receptors have overlapping or redundant functions, the apparent absence of *NLRP4, NLRP7*, and *NLRP9* in wolverine, ermine and European river otter might be duly compensated by other family members. It is not unexpected for gene families to gain or lose members in different species under selective pressure (Helsen *et al.* 2020). To this end, no orthologs for *NLRP7* and *NLRP11* have been identified in the house mouse. In another example, we were not able to identify the wolverine orthologs for *CLEC4A*, *CLEC4C*, and *CLEC4M* (*CLEC4M* is also known as *CD299* or *L-SIGN*). These genes are members of the C-type lectin family of receptors to which the human orthologs recognize glycan moieties on pathogens. Similar to the wolverine, murine orthologs to *CLEC4A*, *CLEC4C* and *CLEC4M*, have not been reported. It should be noted that the aforementioned *NLRP*- and *CLEC4*- genes are not in the current mammalian BUSCO gene set, presumably because of their non-universal representation across mammalian species.

### Viral diseases, innate immunity, and the interferons

Viral diseases are particularly prominent from environmental disruption (Jimenez-Clavero 2012; Cohen *et al.* 2020). An important arm of innate immunity against viral diseases is the interferons, which comprised families of cytokines with antiviral properties as well as pleiotropic activities that include cell proliferation and immunoregulation. Typically, expression of the interferons triggered by viral nucleic acids is induced through the Toll-like receptors or RIG-I members such as *DHX58* (*RIGI*) or *IFIH1* (*MDA5*), and through the appropriate adaptors and signaling transducers (review McNab *et al.* 2015). Table 3 and Supplementary Table 2 provide annotations for wolverine interferons, interferon receptors, and pathway intermediaries.

All interferons have inherent antiviral activities. The interferons and their receptors have radiated to all the vertebrates (reviews Secombes and Zou 2017; Kak *et al.* 2018; Mesev *et al.* 2019). The interferons are classified by the type of receptor subunits through which they signal. The type I interferons include IFN-alpha, IFN-beta, IFN-epsilon, IFN-kappa, and IFN-omega, and signal through the receptor subunits IFNAR1 and IFNAR2. The type II interferon, IFN-gamma, signals through the IFNGR1 and IFNGR2 receptor chains. Type III interferons comprise three members, IFN-lambda1 (IFNL1, Interleukin-29), IFN-lambda2 (IFNL2, Interleukin-28A), and IFN-lambda3 (IFNL3, Interleukin-28B), and signal through a receptor comprising the IFNLR1 and the IL10RB receptor chains. We observed wolverine type III interferon receptor, *IFNLR1*, may utilize a “CTG” codon for translation initiation. All 14 Illumina reads, derived from a PCR-free library, support a nonallelic “CTG” initiation codon. This codon is also supported by the short-read assembly reported for the Eurasian wolverine (Ekblom *et al.* 2018) (Gulo_2.2_annotated; contig CYRY02009887.1). Typically, a vast majority of genes uses an ATG codon for translation initiation; however, translation initiation from noncanonical initiation codons is observed with increasing frequency, although at a much lower rate (review Kearse and Wilusz 2017).

**Fig. 7.**
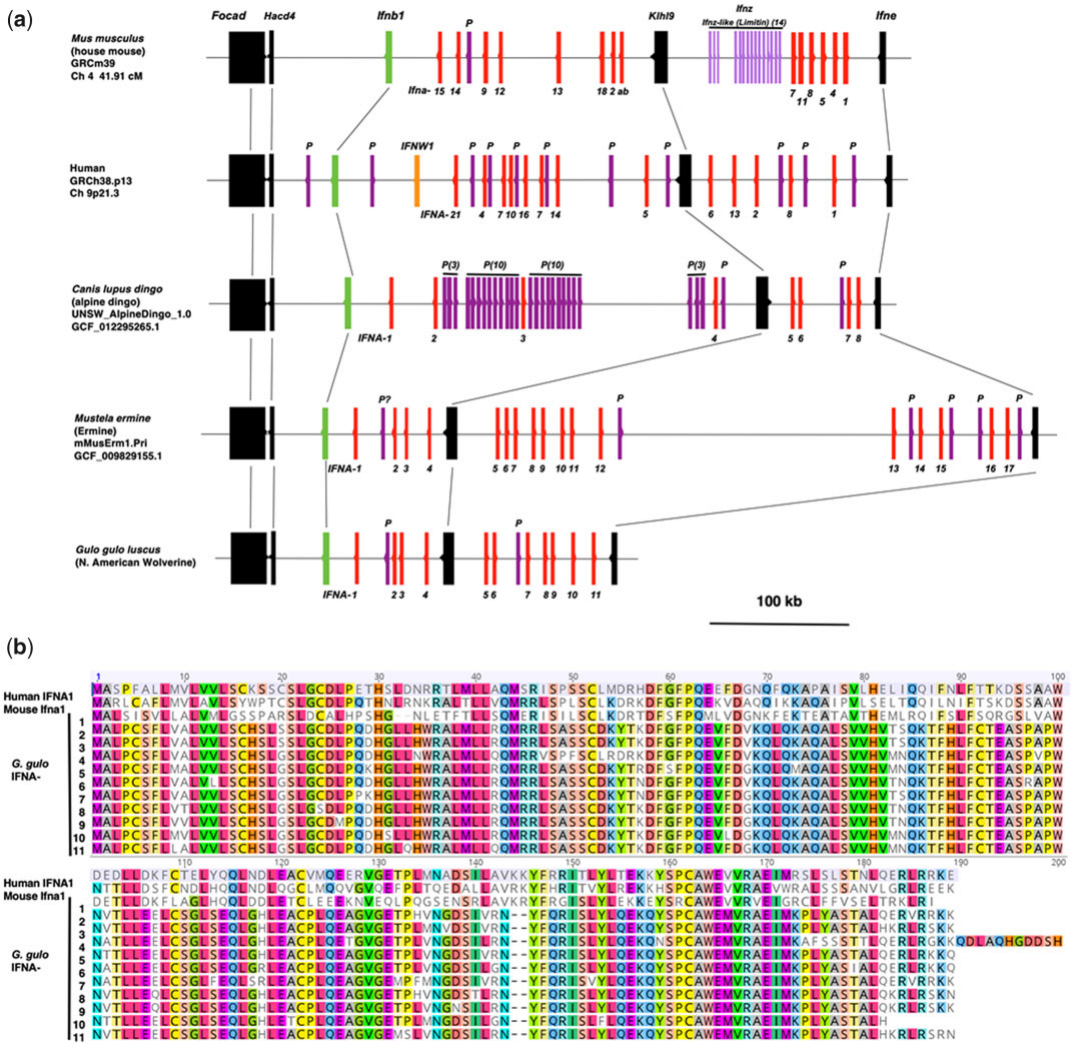
Alignment of interferon alpha/beta gene cluster. a) Structure of wolverine, ermine, dingo, mouse, and human interferon alpha/beta gene clusters. The outdent denotes the direction of transcription. P denotes pseudogene. Human and mouse interferon alpha genes are indicated by their formal assigned gene symbols. Since formal gene symbols have not been assigned for the interferon alpha genes in dingo, ermine, and the wolverine; they are numbered sequentially from left to right in this report. Accessions for the predicted wolverine orthologs are provided in parentheses: *FOCAD* (OM350522); *HACD4* (OM350524); *IFNB1* (OM291625); *IFN1* through *IFN11* (OM350528 through OM350538, respectively); *KLHL9* (OM350543)*; IFNE* (OM350539). b) Wolverine interferon alpha polypeptides aligned to human IFNA1 and mouse Infa1.

In vertebrates, most interferon and interferon receptor genes are organized into two clusters in the genome. The wolverine genes proved to be no exception. In human, the IFN-beta *(IFNB1)*, IFN-alpha *(IFNAs)*, IFN-omega *(IFNW1)*, and IFN-epsilon *(IFNE)* genes reside in a tight cluster on chromosome 9p21.3. The orthologous cluster in wolverine, compared with the human, mouse, dingo (dog), and ermine clusters is depicted in Fig. 7a. As indicated by the flanking genes (*FOCAD, HACD4*, and *KLHL9)*, this region of the wolverine genome is syntenic amongst the five species depicted, with *IFNB1* being the first interferon gene in the cluster, and *IFNE* the last. However, there are notable species-specific differences in cluster composition. Interferon-zeta (*INFZ*) and zeta-like genes are specific to rodents and are not found in the clusters of the other species. Interferon-omega gene (*IFNW*) is human specific and is absent in mouse, dingo, ermine, and the wolverine.

Most notable is the interspecies copy number variations in the interferon-alpha genes, which range from 17 in ermine, 14 in mouse, 13 in human, 10 in wolverine, and 9 in the dingo. The dingo has the smallest number of potentially functional interferon-alpha genes but the highest number of interferon-alpha pseudogenes, at 28. The biological significance of variable numbers of interferon-alpha genes in different species is not clear. One possibility is that individual genes are under differential transcription control, allowing more finely tuned regulation of interferon-alpha levels in different cells and in response to different environmental cues. However, interferon-alpha polypeptides are nearly identical (Fig. 7b) and are believed to signal through the same receptor, suggesting that gene copy number could simply reflect the dosage needed by a given species. In contrast to the interferon-alphas, *IFNB1, IFNE*, and *IFNK* are all single-copy genes in the five species.

Of the six genes encoding chains for the three interferon receptors, four are clustered together on human chromosome 21q22.11. A similar organization is found in the mouse, dingo, ermine, and the wolverine genomes (Supplementary Fig. 3). This cluster comprises genes encoding both chains of the type I interferon receptor, *IFNAR1* (OM291623) and *IFNAR2* (OM291624), one of the two chains of the type II interferon receptor *IFNGR2* (OM291627) and the type III interferon receptor *IL10RB* (OM291646). This gene cluster reveals a high degree of synteny in the five species with respect to gene order, orientation, and separation distance. The genes for the remaining two receptor chains, *IFNGR1* (OM291626) and *IFNLR1* (OM291632), are situated alone and elsewhere in the genome.

### Potential pseudogenes

In addition to *SLC10A5* described earlier in this report, we encountered four other examples of what could be a pseudogene in place of an otherwise functional single copy gene. We observed an in-frame termination codon in Nod-like receptors 2 (*NLRP2*, OM569642) and 14 (*NLRP14*, OM569643), and in Interferon-epsilon (*IFNE*, OM350539). We also observed an internal insertion of a single base (cytosine), which alters the reading frame of Toll-like receptor 10 (*TLR10*, OM291796) when compared with human *TLR10*. We do not believe these four examples are the results of sequencing errors or are allelic variations in our specimen since we observed a complete concordance with our mapped long-reads and Illumina short-reads with a tiling depth of at least 15 across these positions. Moreover, the identical presumptive lesions were also found in the short-read assembly reported for the Eurasian wolverine (Ekblom *et al.* 2018) (Gulo_2.2_annotated) on contigs, CYRY02008074.1, CYRY02019775.1, CYRY02008739.1, and CYRY02043033.1, for *IFNE*, *TLR10*, *NLRP2*, and *NLRP14*, respectively. The aforementioned genes are members of multigene families whose members have overlapping or possibly redundant functions, and is therefore plausible these genes could be subject to loss from adaptive pressure in the wolverine (Helsen *et al.* 2020). Interestingly, a recent report revealed a surprising portion of the human population with inborn errors in genes of the innate immune system, and having no discernible clinical presentation until challenged by a specific pathogen (Zhang *et al.* 2020). Final confirmation whether *IFNE*, *TLR10*, *NLRP2*, and *NLRP14* are indeed nonfunctional pseudogenes in the wolverine or are inborn errors specific to our individual, will require re-sequencing other wolverine specimens.

### Wolverine susceptibility to coronaviruses

Coronaviruses are well known for their ability to cross host-species barriers and for zoonotic and anthroponotic transmissions. The seven known coronavirus strains infecting humans [human coronavirus-NL63 (HCoV-NL63), human coronavirus-NL229E (HCoV-NL229E), human coronavirus-OC43 (HCoV-OC42), human coronavirus-Hong Kong University 1 (HCoV-HKU1), Middle East respiratory syndrome coronavirus (MERS-CoV), severe acute respiratory syndrome coronavirus (SARS-CoV), and severe acute respiratory syndrome coronavirus 2 (SARS-CoV-2)] are believed to have crossed into humans from an animal reservoir. Cross-species transmissions also have veterinary implications, exemplified by swine acute diarrhea syndrome coronavirus (SADS-CoV), which is thought to be bat derived, and the alpaca coronavirus, which is closely related to human 229E virus, suggesting a zoonotic or anthroponotic transmission between humans and alpacas (reviews Li 2015; Millet *et al.* 2020).

Susceptibility to pathogens is an important part of an integrated genomics-based management strategy for the wolverine. Many Mustelidae are susceptible to coronaviruses (Singh *et al.* 2020; Stout *et al.* 2021) as shown by the recent outbreaks from SARS-CoV-2 in high density mink farms (Pomorska-Mól *et al*. 2021). Although ferrets (*Mustela putorius furo*) have been experimentally infected with SARS-CoV-2 (Kim *et al.* 2020), little is presently known about the modality of coronavirus disease transmission in the wild where the population density is lower. Thus far, there are no reported cases of coronavirus infection in wolverine; however, the recent reports of SARS-CoV-2 in wild white-tailed deer (Chandler *et al.* 2021) and in zoo animals reinforce the threat of spillover infection to naïve wildlife (Franklin and Bevins 2020; Delahay *et al.* 2021). Low population density of wolverines makes direct wolverine to wolverine transmission of coronavirus infection unlikely; however, potential infection from prey or scavenged carcasses is a concern. The recent spillover infection into the white-tail deer population would not likely affect the wolverine, since their ranges do not overlap; however, that cannot be said about caribou or moose should SARS-CoV-2 spillover in those and other prey species. To lessen the risk of spillover infection, some wolverine investigators are now testing themselves for COVID-19 and implementing infection prevention protocols when handling wolverines in the field. Hence, it is important to assess the potential susceptibility of wolverine to the coronaviruses from the genomics information in hand.

**Fig. 8.**
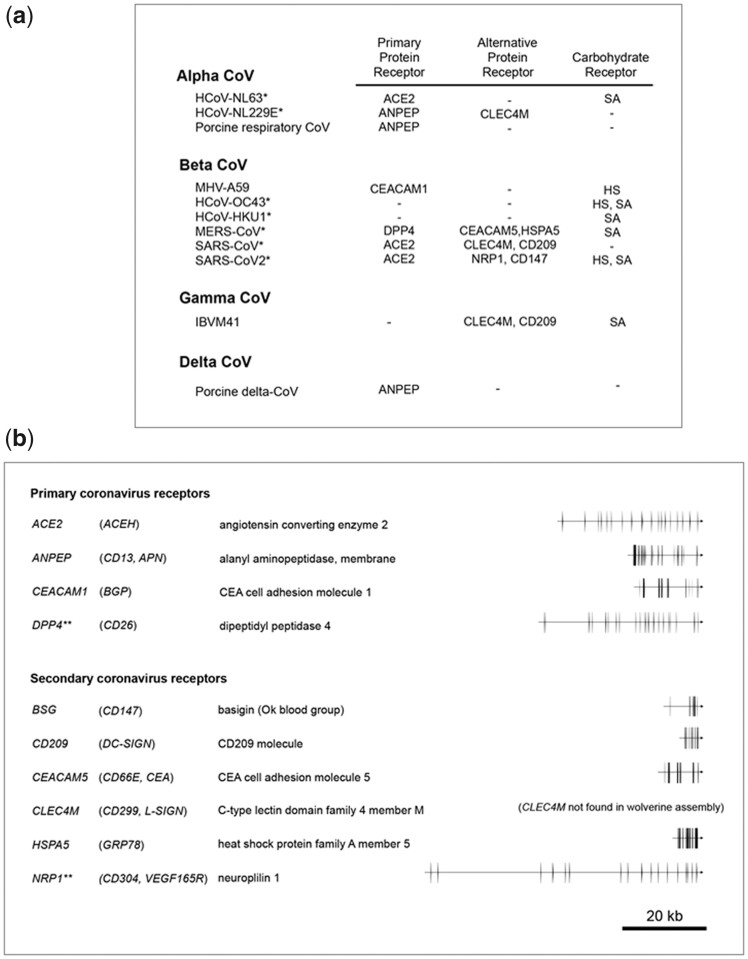
Coronavirus receptor usage. a) Receptor usage by representative members of the four genera of coronavirus. Host cell receptors for virus entry are denoted by their approved gene symbols. The seven known coronaviruses that infect human are indicated by an asterisk. Abbreviations used: HS, heparan sulfate; SA, sialic acid. Figure 8a was adapted from Millet *et al.* (2020). b) Wolverine orthologs of coronavirus entry receptor genes. Genes are denoted by their approved gene symbols, with alternative name(s) in parenthesis. Gene structures are diagrammatically depicted with exons represented by rectangular boxes with the arrow denoting the direction of transcription. Size of the genes is indicted by the 20 kb scale bar. Longer genes, denoted by double asterisks (**), are shown in half scale compared with the other genes. Accessions for predicted wolverine orthologs are provided in parentheses: *ACE2* (OM350479); *ANPEP* (OM569630); *CEACAM1* (OM569632); *DPP4* (OM569639); *BSG* (OM569631); *CD209* (OM291570); *CEACAM5* (OM794715); *CLEC3M* (not found in wolverine assembly); *HSPA5* (OM569641); and *NRP1* (OM569644).

Viral tropism operates at many physiological levels, but viral entry into cells is an important and necessary step. Typically, cell entry is a receptor-mediated process to which the coronaviruses are uniquely adapted to cross species-barrier and to infect multiple cell types within the host (Li 2015; Millet *et al.* 2020). Figure 8a illustrates the large repertoire of different known host genes that could be recruited as an entry receptor by the viral envelope glycoproteins (S-proteins) of different clades of coronaviruses. The ability for the coronaviruses to use one of four different high-affinity primary receptors and any of the five known low affinity secondary receptors, increases the likelihood of finding a productive combination in a new host. The ability to breach species barriers is further enhanced by the high inherent mutation rates in coronaviruses, by the use of carbohydrate (typically heparin sulfate or sialic acid) as entry receptors by some coronaviruses, and by having multiple receptor binding sites on the S-protein, where binding avidity could help offset suboptimal binding in a potential new host.

Figure 8b provides Accessions for predicted wolverine orthologs to the known coronavirus primary and alternative entry receptors. Comparisons of these receptors to those in permissive host species should provide a first insight to the susceptibility of the wolverine to coronaviruses. Since only the contact points between the human S-protein of SARS-CoV-2 and its primary receptor, ACE2, have been mapped to an amino acid resolution, analysis is directed at this particular coronavirus. In human or hamster, at least 31 contact residues between ACE2 and the S-protein of SARS-CoV-2 have been identified using modeling (Chan *et al.* 2020), crystallography (Wang *et al.* 2020), and cryo-electron microscopy (Yan *et al.* 2020). Eight of these 31 positions (human ACE2 residues 24, 30, 34, 41, 82, 83, 353, and 357) are identified by all three techniques used. While it has not yet been shown experimentally, it has been suggested that these contact residues are important for viral binding and cell entry. Since a determinant of SARS-CoV-2 tropism across species is at the receptor level (Murgolo *et al.* 2021), we wish to compare the contact residues identified in human and hamster ACE2 with the analogous residues in Mustelidae. ACE2 is conserved across species with highly conserved blocks along the length of the protein, particularly at or near the S-protein contact residues identified in human and hamster. It is therefore likely that ACE2 is similarly folded across species and that cross-species alignment could be used to infer the presumptive contact residues between mustelid ACE2 and the SARS-CoV-2 S-protein.

**Fig. 9.**
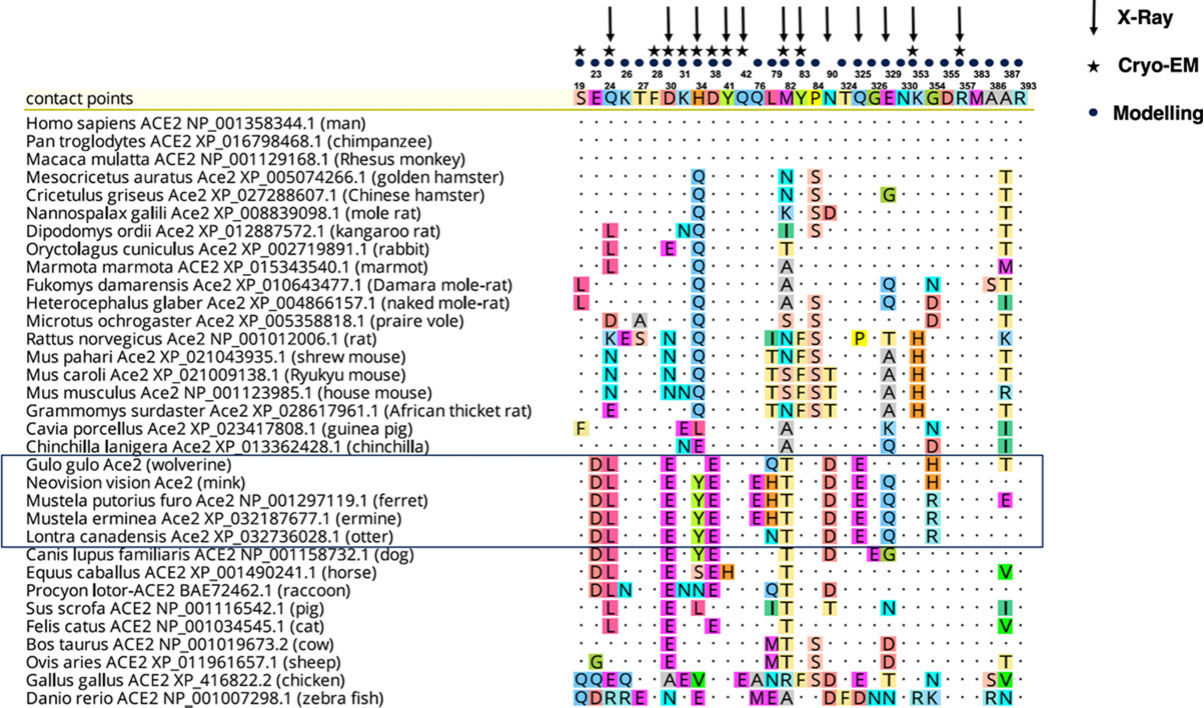
Multiple species alignment of the presumptive contact residues for ACE2 and SARS-CoV-2 S-protein. The contact amino acids in human ACE2 identified by modeling (Chan *et al.* 2020), crystallography (Wang *et al.* 2020), and cryo-electron microscopy (Yan *et al.* 2020), are denoted by arrow, star, and circle, respectively. The corresponding positions in ACE2 for the species indicated (see text) are aligned to the human contact residues. Amino acid contacts identical to human are denoted by dots. The presumptive contact residues for selective Mustelidea, including wolverine, are boxed.

A multiple alignment of the presumptive contact residues for ACE2 and SARS-CoV-2 S-protein of selected species is shown in Fig. 9. Human, chimpanzee, and rhesus monkey ACE2 shared the identical 31 predicted contact residues identified by modeling (Chan *et al.* 2020), crystallography (Wang *et al.* 2020), and cryo-electron microscopy (Yan *et al.* 2020). The golden hamster (*Mesocrgicetus auratus*), an animal model for SARS-CoV-2 infection, deviates at only four positions (Q34, N82, S84, T387) (Chan *et al.* 2020) relative to human ACE2 (Chan *et al.* 2020; Wang *et al.* 2020; Yan *et al.* 2020). Mustelid ACE2 sequences (Fig. 9, boxed), share 18 contact residues in common with human ACE2 (S19, K26, T27, F28, K31, Y41, Q42, Y83, P84, T324, G326, N330, K353, D355, R357, M383, A386, and R393), but have a common core of seven residues (D23, L24, E30, E38, T82, D90, and E325) that are markedly different to rodents and primates. In addition to these seven core residues, the mink (*Neovision vision*) has five additional deviations relative to the primates (Y34, E76, H79, Q329, and H354). Damas *et al.* (2020) recently predicted the host range of SARS-CoV-2 by the conservation properties of 25 presumptive contact residues of ACE2 and S-protein for over 400 vertebrate species. In their analysis the Mustelidae were deemed to be of “very low” risk. However, this finding is contradicted by the infection by SARS-CoV-2 in farmed minks and experimentally in ferrets (*Mustela putorius furo*), indicating viral tropism is complex and that ACE2 interactions alone might not be entirely predictive. At face value, mink and ferret, which are both susceptible to SARS-CoV-2, differed by only two presumptive ACE2 contact residues (Fig. 9, boxed). Although there have not been reports of SARS-CoV-2 infection in ermine, the near-identical contact residues shared with the ferret and mink suggest the ermine is susceptible. The contact residues in wolverine ACE2 appear to be the most different (H34, Q76, Q79, E329, and T387) from the rest of the mustelids. Whether these differences mean the wolverine could be less susceptible to SARS-CoV-2 than the ferret and mink is not clear. However, it should be noted the contact residues for the mustelids are only inferred from sequence alignment with human ACE2, and none have yet been experimentally evaluated.

**Fig. 10.**
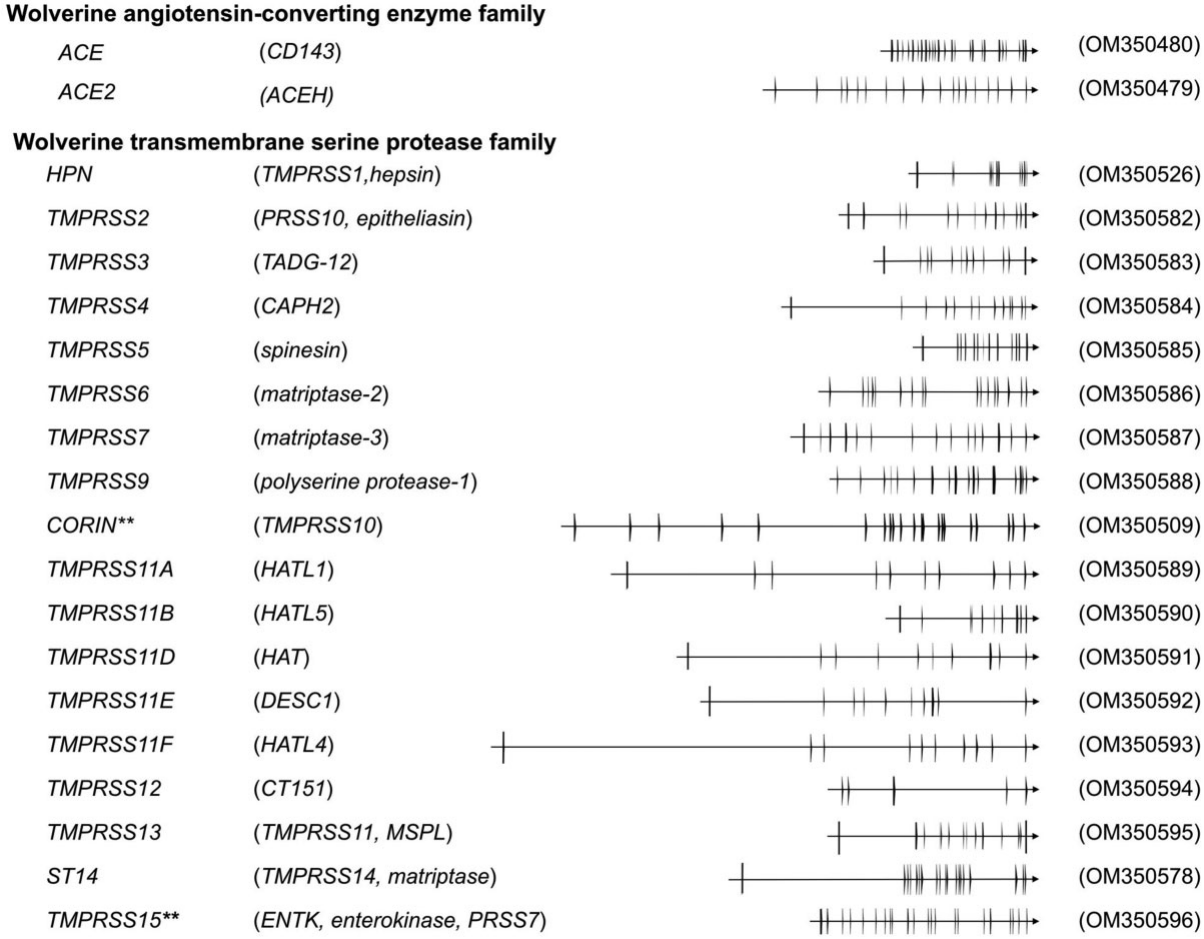
The structure of angiotensin-converting enzyme (*ACE)* and transmembrane serine protease (*TMPRSS)* families of genes in wolverine. Genes are designated by their formal gene symbols with their alternative gene names provided in parentheses. Exons are denoted by vertical bars with the direction of transcription indicated by the arrow. Size of the genes is indicated by the 10-kb scale bar. Longer genes, denoted by double asterisks (**), are shown in quarter scale compared with the other genes. Accessions for predicted wolverine orthologs are provided in parentheses.

In addition to different cell surface receptors, entry of SARS-CoV-2 into cells requires a second host factor, a transmembrane serine protease (TMPRSS2), which processes the viral surface S protein to a form that can be presented and recognized by the host cell receptors (Hoffmann *et al.* 2020). In human, *TMPRSS2* is a member of a multigene family comprising the 18 known type II membrane-anchored serine proteases (review Antalis and Buzza 2016). All 18 members are complete in our wolverine assembly. Although *TMPRSS2* has so far been shown to be primarily responsible for SARS-CoV-2 entry into cells, other *TMPRSS* members, notably, *TMPRSS4*, *TMPRSS11A*, *TMPRSS11D*, and *HPN* (*TMPRSS1)*, have been implicated to act in synergy or in place of *TMPRSS2* for enhanced infectivity and systemic COVID-19 infection (Kishimoto *et al.* 2021; Fuentes-Prior 2021). It is therefore likely that processing of the S-protein by the different cell surface serine proteases could have quantitative or qualitative effects influencing tropism. As a resource to study the role of these different serum proteinases in viral infection, annotation of the wolverine *TMPRSS* family is provided in Fig. 10.

### Assessment of genetic diversity

From a single wolverine specimen, our assessment of genetic diversity is limited thus far to the quantification of heterozygous SNVs and indels. Larger insertions or deletions, typically termed structural variants, were excluded in the present study since they are far less abundant and cannot be efficiently mapped and tabulated using Illumina short-reads. Although the heterozygosity of SNVs and indels from a single animal provides an incomplete picture of the species’ genetic diversity, a recent report shows it could be a surprisingly good predictor of survival in the restoration of threatened desert tortoise (Scott *et al*. 2020).

**Fig. 11.**
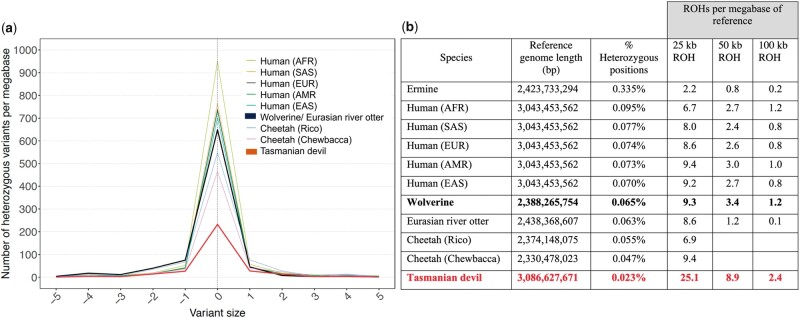
Heterozygous SNV and indel frequencies in wolverine. a) Heterozygous SNV and indel frequencies in wolverine are plotted against those of a diverse outbred population (human), Eurasian river otter, and two species that had underwent recent genetic bottleneck, the cheetah, and the Tasmanian devil. At the scale shown, the plots for the wolverine and the Eurasian river otter are superimposable. Heterozygous SNV level for the ermine is nearly 5-fold higher (0.33%) compared with the wolverine (0.065%), and is not plotted. Heterozygous SNVs are denoted by a variant size of zero on the plot. Negative and positive values on the *X*-axis denote the size of heterozygous deletions and insertions (bp), respectively. For ease of comparisons, variant frequencies are normalized to 1 million-base in the genome. b) Comparison of Runs of homozygosity (ROH) of wolverine against ermine, Eurasian river otter, human, cheetah, and Tasmanian devil (see *Materials and methods*). Wolverine and Tasmanian devil ROH values are highlighted in bold. Abbreviations: AFR, African; AMR, Admixed American; EAS, East Asian; EUR, European; ROH, run of homozygosity; SAS, South Asian.

Figure 11 compares the normalized heterozygous variants for the wolverine genome against the ermine, Eurasian river otter, representative members of a diverse outbred population (human), and individuals of two animal populations that had undergone recent genetic bottlenecks, the cheetah (*Actinonyx jubatus*) (Menott-Raymond and O’Brien 1993), and the Tasmanian devil (*Sarcophilus harrisii*) (Miller *et al.* 2011). SNVs are represented by a variant size of 0, negative, and positive numeric values represent sizes of deletions and insertions in base pairs, respectively. SNVs are by far the most abundant type of variants and will be the main focus of analysis. The degrees of heterozygosity, expressed as a percentage of the number of heterozygous SNVs to the estimated size of the respective genomes, are tabulated in Fig. 11b.

Our wolverine genome has a heterozygosity SNV level of 0.065%, which is nearly identical to the 0.063% value calculated for the Eurasian river otter, marginally below the 0.070% (East Asian) to 0.095% (African) values calculated for the five major human ethnic populations, and is markedly higher than the 0.023% value for the Tasmanian devil, where reduced genetic diversity is believed to be a major contributing factor to devil facial tumor disease, a transmissible cancer (Epstein *et al.* 2016; Storfer *et al.* 2017). Deleterious traits have been attributed to the reduced genetic diversity in the cheetah (O’Brien *et al.*^1985^, ^2017^), although a recent report has shown clear genetic differentiations between different cheetah subspecies, refuting earlier assumptions that cheetahs showed little population differentiation (Prost *et al.* 2020). Quite possibly, genetic diversity in cheetah populations might be higher than previously thought. At least from single specimens, our tabulation of heterozygous SNVs for the cheetah showed an intermediate value between that of the wolverine and the Tasmanian devil. We calculated a heterozygous SNV value of 0.047% for Namibian cheetah Chewbacca. This value is significantly higher than 0.021% that was previously reported for the same animal (Dobrynin *et al.* 2015). Our calculated value of 0.055% agrees with the published result of 0.050% for Rico, a second Namibian cheetah (Tamazian *et al.* 2021), and for the 0.023% value previously reported for Tasmanian devil ERS3900573 (Dobrynin *et al.* 2015). Interestingly, heterozygosity SNV level calculated for this ermine specimen is nearly 5-fold higher (0.33%) compared with the wolverine (0.065%) and the Eurasian river otter (0.063%). We do not know why this ermine genome (mMusErm1.Pri) is so diverse in respect to SNV heterozygosity. This particular ermine (BioSample SAMN12611999) is a male specimen collected in New Zealand, and is designated as an invasive species primarily founded from stock caught in Lincolnshire UK and introduced to New Zealand in the 1880s and 1890s.

We then examined the gross arrangements of the heterozygous SNVs in human, wolverine, and Tasmanian devil. Specifically, we tabulated the so termed ROH, which is the average number of homozygous stretches of 25, 50, or 100 kb lengths per mega-base of genome that are free of heterozygous SNVs. The cheetahs were excluded from this analysis at the 50 and 100 kb intervals because the cheetah assemblies were too fragmented to provide a meaningful assessment of long homozygous stretches. As shown in Fig. 11b, for the three intervals tested, ROH in the wolverine is only slightly greater (less diverse) than the human population, and much less (more diverse) than the Tasmanian devil. At least for the 25 kb interval, ROHs for the cheetahs are not too dissimilar to wolverine or to the general human population, with Namibian cheetah Chewbacca less diverse than Namibian cheetah Rico. At face value from a single sample, the ROH values at 50 and 100 kb for the ermine and Eurasian river otter is less (more diverse) than the wolverine.

Although our findings are preliminary, based on values in close proximity with human, the results are suggestive of a relatively diverse genetic pool for our archival wolverine specimen from Nunavut collected 30 years ago, and will serve as a useful historical baseline to support the resequencing of contemporary wolverines now underway in our laboratory.

### Conservation genomics and the wolverine

Conservation genomics is an integrated collection of genomics technologies, methodologies, and multidisciplinary expertise, to study and stem the worldwide loss of biodiversity (reviews Khan *et al.* 2016; Supple and Shapiro 2018; Wright *et al.* 2020; Hohenlohe *et al.* 2021). High-quality reference genomes are the mainstays of conservation genomics, providing an understanding of the distribution and functional significance of genetic variations in natural populations in response to the changing environment (Brandies *et al.* 2019). The long-read assembly of the wolverine presented in this study is an improvement over the short-read assembly representing this species, and is the third chromosomal-level assembly for Mustelidae.

At a base level for conservation and management, the wolverine reference genome presented here will provide a benchmark of genetic diversity that can be compared with past and future samples across its host range, as well as enabling a comparison of the genomic differences between the Eurasian and North American wolverines. In this context, the assembly represents a resource for future genomic marker development for the North American wolverine, notably through whole genome resequencing, which provides the highest resolution and sensitivity for variant discovery. Resequencing would complement current reduced-representation sequencing efforts, such as Rad-Seq and related methods (Andrew *et al.* 2016). It should be noted that the role of genomic diversity in species conservation is complex and evolving. The traditional paradigm that focuses on maximizing genetic diversity in susceptible populations is giving way to alternative management strategies of minimizing deleterious variations (Kyriazis *et al.* 2021). As shown by the recent genome sequencing of the Kakapo genomes, a small highly inbred population can still be relatively healthy in the absence of deleterious alleles (Dussex *et al.* 2021).

The importance of neutral and adaptive markers in molecular ecology has been reviewed by Kirk and Freeland (2011). SNVs have provided increased power to estimate genetic differentiation in wildlife populations affected by anthropogenic factors (Trumbo *et al.* 2019). The availability of thousands of new SNVs for wolverine populations through whole genome sequencing will improve estimates of gene flow, inbreeding, relatedness, parentage and effective population size, and the timing of past extirpation and recolonization in their southern range and decline in their eastern range. These are critical parameters for this highly vagile species whose populations are becoming increasingly small and isolated (Väli *et al.* 2008; Ekblom *et al.* 2021). Finally, identifying loci under selection (Bay *et al.* 2018; Waterhouse *et al.* 2018) could give a first look at local and future adaptive potential or vulnerability across the wolverine’s North American range.

While the cost of DNA sequencing has decreased dramatically, it is currently not low enough for routine direct screening of large cohorts outside medicine, where whole genome resequencing of patients is becoming the norm (Costain *et al.* 2021). In the immediate future, the use of resequencing in conservation genomics is likely to be the first part of a two-step process, where it will be first used to tabulate variants from small informative cohorts, from which informative and low-cost genotyping panels could be constructed to carry out the main screen. Such a genotyping panel was recently developed for the Eurasian wolverine, where a 96-marker SNV set was shown to outperform the previously used 19-microsatellite (SSR) panel (Ekblom *et al.* 2018, 2021). A comprehensive SNV panel for the North American wolverine populations would enable highly efficient and cost-effective genotyping of hundreds to thousands of neutral and adaptive SNV markers from archival and noninvasively collected field samples. Supported by our reference genome, re-sequencing of selected wolverines is underway to develop this resource.

## Data availability

Sequences described for the North America wolverine are submitted to GenBank and other public archives and repositories. This study is assigned BioProject numbers PRJNA837618, PRJNA675847, and PRJNA775072 to facilitate data dissemination. Wolverine genome assemblies, Gulo_gulo_luscus_A-V1.0, Gulo_gulo_luscus_F-V1.0, and Gulo_gulo_luscus_R-V1.0 are assigned WGS Accessions: JAMKPV000000000, JAJAGD000000000, and JAJHUB000000000, respectively. Accessions and supplementary information for wolverine genes depicted in the manuscript are found in Table 3 and Supplementary Table 2.

Supplemental material is available at *G3* online.

## Supplementary Material

jkac138_Supplemental_legends

jkac138_Table_S1

jkac138_Table_S2

jkac138_Figure_S1

jkac138_Figure_S2

jkac138_Figure_S3
