# Supplementary material for: Chromosomal-level reference genome assembly of the North American wolverine (Gulo gulo luscus): a resource for conservation genomics
Source: G3 (Bethesda). 2022 Jun 8;12(8):jkac138. doi: 10.1093/g3journal/jkac138 (PMC9339297; doi:10.1093/g3journal/jkac138)
Supplement: jkac138_Supplemental_legends [file jkac138_supplemental_legends.docx]

**Supplementary Figure 1**

Contamination assessment of the wolverine Gulo_gulo_luscus_F-V1.0 assembly (JAJAGD000000000). The assembly was divided into 478,025 non-overlapping 5 kb segments. Each segment was searched using BLAST (Altschul *et al*. 1990) against the non-redundant NCBI Nucleotide Database (Version: 2020 Nov 19) (https://ftp.ncbi.nlm.nih.gov/blast/db/). The resulting BLAST results were tabulated using a series of five sequential filters (indicated by red circles), of decreasing phylogenetic distance from the wolverine: (1) The genome assembly of the ermine (*Mustela erminea*; GCF_009829155.1) and the Eurasian river otter (*Lutra lutra*; GCA_902655055.2); (2) Other members of Mustelidae; (3) non-Mustelidae carnivores; (4) organisms outside of carnivora; and (5) a collection of 11 individual NCBI taxonomic lineages (See MATERIALS AND METHODS).

**Supplementary Figure 2**

Wolverine mitochondrial genome. (A) Annotated map of the North American wolverine mitochondrial genome (JAJAGD000000000; contig WOV01_MT20201101). In accordance to the usual convention, genes are annotated clockwise from the transcription start site of the *tRNA Phe* gene, denoted as position 1. (B) Short read support for the mitochondrial genome assembly (See MATERIALS AND METHODS). (C) Phylogenetic neighbour-joining tree of mitochondrial genomes of selective members of Mustelidae. Tree was generated using Geneious 10.2.6 (Biomatters, Auckland, NZ).

**Supplementary Figure 3**

Interferon receptor gene cluster. (A) Human, mouse, and wolverine *IFNAR1*, *IFNAR2*, *IL10RB,* and *IFNGR2* share a common organization in the three species. Interferon receptor genes are depicted by red boxes. Flanking genes syntenic in the three species are depicted by black boxes. Outdents denote the direction of transcription. (B) Receptor gene structure. Exons are depicted by red rectangular boxes with the arrow denoting the direction of transcription. Accessions for wolverine genes: *OLIG2* (OM569646); *OLIG1* (OM569645); *IFNAR2* (OM291624); *IL10RB* (OM291646); *IFNAR1* (OM291623); *IFNGR2* (OM291627); *TMEM50B* (OM569653); *DNAJC28* (OM569637); *GART* (OM569640); *SON* (OM569649), *DONSON* (OM569638); *CRYZL1* (OM350510). Accessions for predicted wolverine orthologs are provided in parentheses.

**Supplementary Table 1**

Tabulation of the 169 reported mustelid microsatellite loci and their status in the Gulo_gulo_luscus_F-V1.0 assembly.

**Supplementary Table 2**

Annotated genes and accessions for wolverine genes depicted in the present study. Genes are organized by tabs.
